# Organ Weights in *NPC1* Mutant Mice Partly Normalized by Various Pharmacological Treatment Approaches

**DOI:** 10.3390/ijms24010573

**Published:** 2022-12-29

**Authors:** Veronica Antipova, Lisa-Marie Steinhoff, Carsten Holzmann, Arndt Rolfs, Carlos Junior Hempel, Martin Witt, Andreas Wree

**Affiliations:** 1 Institute of Anatomy, Rostock University Medical Center, D-18057 Rostock, Germany; 2 Gottfried Schatz Research Center for Cell Signaling, Metabolism and Aging, Macroscopic and Clinical Anatomy, Medical University of Graz, A-8010 Graz, Austria; 3 Institute of Medical Genetics, Rostock University Medical Center, D-18057 Rostock, Germany; 4 Centre of Transdisciplinary Neuroscience Rostock, D-18147 Rostock, Germany; 5 Medical Faculty, University of Rostock, D-18055 Rostock, Germany; 6 Department of Anatomy, Technische Universität Dresden, D-01307 Dresden, Germany

**Keywords:** NPC1, lipid storage disorder, treatment effect, miglustat, 2-hydroxypropyl-β-cyclodextrin, allopregnanolone, organ weights, organs dimension, stomach volume, femur length, gender-specific effects

## Abstract

Niemann-Pick Type C1 (NPC1, MIM 257220) is a rare, progressive, lethal, inherited autosomal-recessive endolysosomal storage disease caused by mutations in the *NPC1* leading to intracellular lipid storage. We analyzed mostly not jet known alterations of the weights of 14 different organs in the BALB/cNctr-*Npc1*^m1N^/-J Jackson *Npc1* mice in female and male *Npc1^+/+^* and *Npc1^−/−^* mice under various treatment strategies. Mice were treated with (i) no therapy, (ii) vehicle injection, (iii) a combination of miglustat, allopregnanolone, and 2-hydroxypropyl-ß-cyclodextrin (HPßCD), (iv) miglustat, and (v) HPßCD alone starting at P7 and repeated weekly throughout life. The 12 respective male and female wild-type mice groups were evaluated in parallel. In total, 351 mice (176 *Npc1^+/+^*, 175 *Npc1^−/−^*) were dissected at P65. In both sexes, the body weights of None and Sham *Npc1^−/−^* mice were lower than those of respective *Npc1^+/+^* mice. The influence of the *Npc1* mutation and/or sex on the weights of various organs, however, differed considerably. In males, *Npc1^+/+^* and *Npc1^−/−^* mice had comparable absolute weights of lungs, spleen, and adrenal glands. In *Npc1^−/−^* mice, smaller weights of hearts, livers, kidneys, testes, vesicular, and scent glands were found. In female *Npc1^−/−^* mice, ovaries, and uteri were significantly smaller. In *Npc1^−/−^* mice, relative organ weights, i.e., normalized with body weights, were sex-specifically altered to different extents by the different therapies. The combination of miglustat, allopregnanolone, and the sterol chelator HPßCD partly normalized the weights of more organs than miglustat or HPßCD mono-therapies.

## 1. Introduction

Niemann-Pick Type C1 (NPC1) is a rare, progressive, lethal, inherited autosomal-recessive endolysosomal storage disease caused by mutations in the *NPC1* gene located on chromosome 18q11 [1,2,3]. A mutation in *NPC1* leads to massive intracellular accumulation of unesterified cholesterol, sphingomyelin, phospholipids, glycosphingolipids, and GM2 and GM3 gangliosides in late endosomes/lysosomes and the Golgi apparatus [4,5,6].

Patients with NPC1 initially present with visceral symptoms such as hepatosplenomegaly, which is followed by progressive neurodegeneration leading to severe motor deficits and various psychiatric and neurological symptoms [7,8,9,10,11,12,13,14,15,16,17,18,19,20,21,22,23].

We used the BALB/cNctr-*Npc1*^m1N^/-J Jackson *Npc1* mouse strain [24,25,26,27,28,29] that carries a spontaneous mutation of *Npc1*. These mice have an almost total absence of the Npc1 protein and display pathological hallmarks of human disease [24,29,30,31,32].

Presently, there is no effective cure for NPC disease [33,34,35], which is able to minimize both general symptoms and neurodegeneration [36,37,38]. In the face of the lack of any causal therapy to date, the iminosugar miglustat (MIGLU) (N-butyldeoxynojirimycin) (Zavesca^®^, Actelion Pharmaceuticals, Allschwil, Switzerland), introduced by Lachmann and Platt in 2001 [39] and acting as a substrate reduction agent, is the only drug approved in Europa, Canada, and Japan to treat the progressive neurological manifestations of NPC1 disease in adults and children [14,40,41].

Miglustat (MIGLU) is a small iminosugar molecule that reversibly inhibits glucosylceramide synthase, the enzyme that catalyzes the first committed step of glycosphingolipid synthesis [42,43,44]. MIGLU is able to cross the blood–brain barrier, allowing it to access malfunctioning neurons in the brain [45]. MIGLU has been shown to delay disease progression and stabilize neurological symptoms in several randomized, controlled clinical trials, observational studies, and long-term extension studies [46,47,48,49,50]. Thus, the depletion of glycosphingolipids by MIGLU reduced pathological lipid storage, improved endosomal uptake, and normalized lipid trafficking in peripheral blood B lymphocytes, improving clinical symptoms [51,52,53]. Furthermore, MIGLU is believed to reduce oxidative stress, and in the course of long-term therapy, it was well tolerated, increased lifespan, and stabilized neurologic functions in NPC patients [54]. However, MIGLU-treated patients complain of different side effects such as weight decrease, abdominal pain, diarrhea, flatulence, and tremors [48,55,56,57]. In the murine and feline models of NPC, MIGLU has been shown to reduce neuronal glycosphingolipid accumulation, delay the onset of neurological dysfunction, and prolong the lifespan of the animals [44,58].

A further seemingly promising drug is the neurosteroid allopregnanolone (ALLO). Griffin et al. [59] showed that adding ALLO to the drinking water only slightly increased the lifespan of *Npc1^−/−^* mice from 67 to 80 d. Concurrently, locomotor function and coordination declined at 8 weeks in both untreated and ALLO-treated *Npc1^−/−^* mice, but the rate of decline was lower in ALLO-treated NPC mice. Interestingly, there was no difference in survival, locomotor function, or motor coordination between male and female mice receiving ALLO treatment. However, replacement therapy with injected allopregnanolone—solubilized in the sterol chelator 2-hydroxypropyl-ß-cyclodextrin (HPßCD)—has been demonstrated to delay the onset of neurological symptoms in the mouse model of NPC1 to increase Purkinje and granule cell survival in the cerebellum, to reduce cortical ganglioside accumulation, cholesterol accumulation, and inflammation and to enhance myelination [59,60,61]. Ahmad et al. [60] confirmed the results of Griffin et al. [62] that a single injection of ALLO solubilized in HPßCD at postnatal day (P) 7 considerably extended the life span of *Npc1^−/−^* mice and additionally showed that injections starting at P7 and repeated at 2-week intervals, had a moderately better outcome than the single P7 injection. Furthermore, ALLO treatment results in the improvement of neurological symptoms in *Npc1^−/−^* mice, significantly reduces microglial activation, and increases neuronal survival. The effects on survival and weight loss of a single injection on P7 followed by injections every 2 weeks were found to be more beneficial than following a single injection at P7 [60].

Davidson et al. [40] described that administration of ALLO solubilized in HPßCD to *Npc1^−/−^* mice at P7 was beneficial; the treated mice exhibited delayed clinical onset, extended lifespan, and reduced ganglioside accumulation. Simultaneously, the same group announced that ALLO without HPßCD does not appear beneficial: administration of HPßCD had the same impact on ameliorating disease progression in *Npc1^−/−^* mice as did the administration of ALLO solubilized in HPßCD [40]. Interestingly, single or multiple doses of HPßCD when administered systemically, improved CNS disease morphology, significantly reduced lysosomal cholesterol accumulation in almost every organ, delayed the progression of neurodegeneration and significantly prolonged the lifespan of *Npc1^−/−^* mice by allowing trapped cholesterol within the late endosome/lysosome to be released [4,40,63,64,65,66,67].

According to Ramirez et al. [68], weekly administration of HPßCD overcomes the lysosomal transport defect associated with the Npc1 mutation, nearly normalizes hepatic and whole-animal cholesterol pools, prevents the development of liver disease and slows down cerebellar neurodegeneration, but has little or no effect on the development of progressive pulmonary disease. HPßCD administration reverses the cholesterol transport defect seen in the *Npc1^−/−^* mice at any age, and this reversal allows the sequestered sterol to be excreted from the body as bile acid [4]. In addition, cyclic oligosaccharides are known to extract cholesterol from the plasma membrane of a variety of cells in vitro [69,70,71]. Therefore, it seems clear that not ALLO but HPßCD alone was responsible for most and possibly for all of the effects of ALLO/HPßCD treatment [59,60,63,64].

Taking the studies together, three drugs were reported to positively affect lifespan and clinical symptoms in *Npc1^−/−^* mice: MIGLU, ALLO, and HPßCD [32,40,60,66,72,73,74,75]. The COMBI treatment, recommended by Davidson et al. [40], has been shown to reduce cerebellar neurodegeneration and intracellular lipid storage, resulting in the prevention of further Purkinje cell loss and an increased lifespan in *Npc1* mutant mice [34,40,54,72,73,74]. Moreover, COMBI therapy positively influenced the motor function of *Npc1^−/−^* mice [73].

NPC1 disease is one of many lysosomal storage diseases and results mainly from a mutation that inactivates the NPC1 protein responsible for the transport of unesterified cholesterol from the late endosomal/lysosomal compartment to the cytosol in every cell [76]. This causes cholesterol to accumulate in all organs and practically in all tissues in the body; the accumulation is characterized by progressive enlargement of the internal organs, causing organ dysfunction, which can manifest clinically as hepatosplenomegaly, splenomegaly, prolonged newborn jaundice, liver dysfunction, lipoid pneumonitis, lung failure and, ultimately, progressive neurological dysfunction secondary to selective neurodegeneration [28,54,68,77,78,79,80,81,82].

With the exception of a recent study [75], most studies on the therapeutic effects of various drugs in *Npc1^−/−^* mice and the respective wild-types were evaluated in gender-mixed groups [37,40,59,73,83,84,85]. Also, clinical observations in NPC1 patients described primarily only age-dependent heterogeneity of the beginning, expression, and symptoms of the disease without differentiation of the patients’ gender [86,87,88,89]. Only the study performed by Walterfang et al. [90] described two siblings with schizophrenia whose adult NPC genotypes were identical but showed dimorphism in their illness courses and their clinical and biochemical parameters due to gender. The authors suggest that the course of the disease and the degree of impairment may be different in female patients and that sex hormones may play a role. However, they stated that human data on the effect of sex on the biochemical and clinical parameters in NPC disease are lacking.

Recently, using a mouse model of NPC1 [75], the therapeutic effects of the COMBI therapy with that of MIGLU or HPßCD alone on body and brain weight and the behavior of *Npc1^−/−^* mice were compared in a larger cohort, with special reference to gender differences. Our results suggest that, in *Npc1^−/−^* mice, each drug treatment regimen had a beneficial effect on at least some of the parameters evaluated when compared to Sham-treated mice, partly showing gender-specific therapeutical benefit.

Although in NPC1 lysosomal storage of various lipids is found throughout the body, until now, the existing few studies in different animal models of NPC1 described only the alterations of some disease-involved organs, including mainly liver, spleen, lung, and brain [28,54,84,91,92,93,94,95,96,97,98] (Supplementary Materials Table S1). Therefore, in the present study, we analyzed, for the first time, alterations of weight and relative weight, dimension, and volume of 14 organs in BALB/cNctr-*Npc1*^m1N^/-J Jackson Npc1 mouse strain in larger cohorts of female and male *Npc1^+/+^* and *Npc1^−/−^* mice. All parameters were evaluated in one trial after COMBI medication as well as after exclusive MIGLU and HPßCD medication in the different groups, each consisting of more than 12 animals and with special reference to gender: male and female *Npc1^−/−^* mutant mice treated with (i) no therapy (None), (ii) vehicle injection (Sham), (iii) combination of MIGLU, ALLO, and HPßCD (COMBI), (iv) MIGLU alone (MIGLU), (v) HPßCD alone starting at P7 and repeated weekly throughout life (HPßCD), and (vi) HPßCD alone given only once at P7 (HPßCD1x). The 12 respective *Npc1^+/+^* mouse (male and female wild-type mice) groups were evaluated in parallel. In addition to the recently evaluated mice (n = 239; [75]), further mice (n = 112) from ongoing experiments were included here. Thus, this study is based on the dissection of 351 mice (176 *Npc1^+/+^*, 175 *Npc1^−/−^*) evaluated in the 24 groups. A total of 6835 organ measurements were included in the study.

Because we did not find noticeable differences in the weights between the left and right sides in any pair of organs in male and female animals, we further analyzed averaged weight values.

## 2. Results

In Table 1, the measures found in the male and female *Npc1^+/+^* and *Npc1^−/−^* mice of the untreated None groups are summarized.

### 2.1. Body Weight

**Figure 1:**
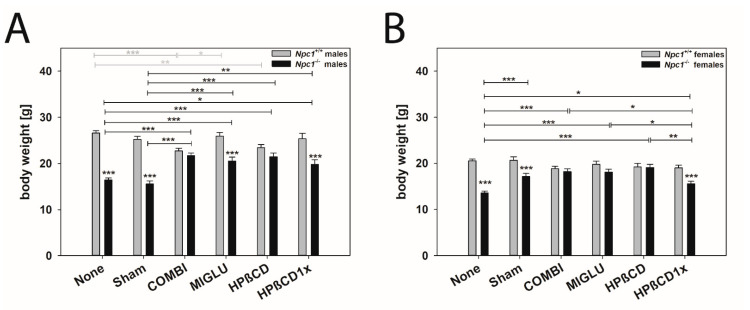
Body weight of male (**A**) and female (**B**) *Npc1* mice. Significant post-hoc tests are indicated by asterisks (* *p* < 0.05, ** *p* < 0.01, *** *p* < 0.001). Data are means ± SEM.

Male mice: The body weights of the male *Npc1^+/+^* mice of the groups None (26.60 ± 0.54 g) and Sham (25.23 ± 0.67 g) did not differ significantly (Figure 1A). The weights of the None (26.60 ± 0.54 g) and MIGLU (25.95 ± 0.74 g) groups were significantly higher than those of the COMBI group (*p* < 0.001 and *p* = 0.01, respectively). Body weights in the HPßCD group (25.95 ± 0.74 g) were lower than those of the None group (*p* = 0.006) (Figure 1A). The body weights of the male *Npc1^−/−^* mice of the None (16.44 ± 0.426 g) and Sham (15.58 ± 0.65 g) groups did not differ significantly (Figure 1A). The body weights of all 4 *Npc1^−/−^* treatment groups significantly increased compared with both None and Sham groups: COMBI (21.75 ± 0.54 g), MIGLU (20.56 ± 0.83 g), HPßCD (21.48 ± 0.83 g) and HPßCD1x (19.80 ± 1.04 g) groups, respectively (Figure 1A). When male wild-type and mutant mice were compared, *Npc1^−/−^* mice had significantly reduced body weights in the None, Sham, MIGLU, and HPßCD1x groups (each *p* < 0.001) (Figure 1A).

Female mice: The body weights of the female *Npc1^+/+^* mice of groups None (20.56 ± 0.36 g), Sham (20.64 ± 0.76 g), COMBI (18.819 ± 0.51 g), MIGLU (19.75 ± 0.72 g), HPßCD (19.22 ± 0.76 g) and HPßCD1x (19.00 ± 0.56 g) did not differ significantly (Figure 1B). In female *Npc1^−/−^* mice, the body weights of the groups Sham (17.13 ± 0.65 g), COMBI (18.18 ± 0.59 g), MIGLU (18.04 ± 0.66 g), HPßCD (19.09 ± 0.68 g) and HPßCD1x (15.54 ± 0.57 g) were increased compared with the None group (13.54 ± 0.45 g). Comparison of female wild-type and mutant mice revealed significantly lower body weights in *Npc1^−/−^* mice of the None, Sham, and HPßCD1x groups (each *p* < 0.001) (Figure 1B). In contrast, the weights of COMBI, MIGLU, and HPßCD groups did not differ significantly (Figure 1B) from wild-types.

### 2.2. Thoracic Organ Weights

#### 2.2.1. Heart

**Figure 2:**
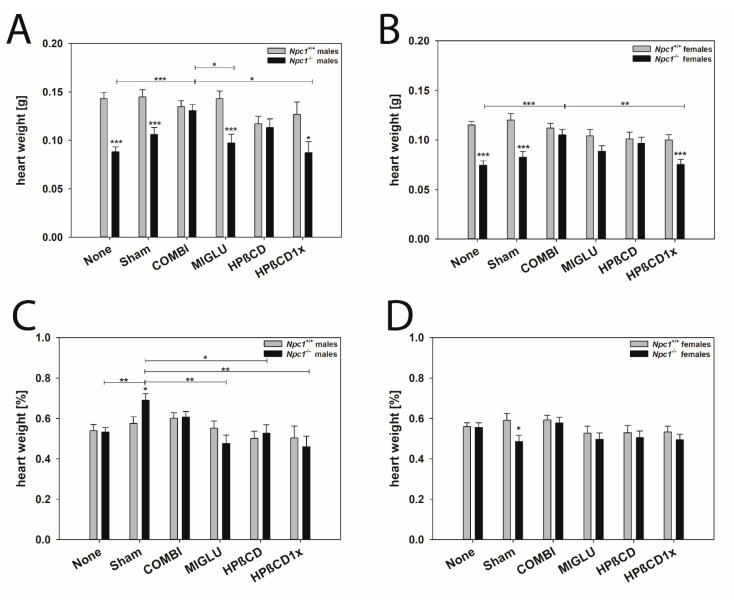
Heart weight (**A**,**B**) and relative heart weight (**C**,**D**) of *Npc1* mice. Male (**A**,**C**) and female (**B**,**D**) mice. Significant post-hoc tests are indicated by asterisks (* *p* < 0.05, ** *p* < 0.01, *** *p* < 0.001). Data are means ± SEM.

Male mice: The heart weights of male *Npc1^+/+^* mice of the groups None (0.143 ± 0.00657 g) and Sham (0.145 ± 0.00735 g) did not differ significantly from those of the groups COMBI (0.135 ± 0.00617 g), MIGLU (0.143 ± 0.00805 g), HPßCD (0.117 ± 0.00767 g), and HPßCD1x (0.127 ± 0.0127 g) (Figure 2A). Likewise, the respective weights of male *Npc1^−/−^* mice of the None (0.0882 ± 0.00509 g) and Sham (0.106 ± 0.00706 g) groups did not differ significantly (Figure 2A). The weights of the COMBI (0.131 ± 0.00600 g) group were significantly higher than in the None (0.0882 ± 0.00509 g), MIGLU (0.0973 ± 0.00900 g), and HPßCD1x (0.0872 ± 0.0114 g) groups (Figure 2A). The heart weights of the male *Npc1^−/−^* mice of the None, Sham, MIGLU, and HPßCD1x groups were significantly lower compared with the respective groups of the *Npc1^+/+^* mice (Figure 2A). The heart weights of the COMBI and HPßCD groups of male *Npc1^−/−^* and *Npc1^+/+^* mice did not differ significantly (Figure 2A).

The relative heart weights (% of body weight) of male *Npc1^+/+^* mice did not differ significantly between all six groups (Figure 2C): None = 0.539 ± 0.0030%, Sham = 0.575 ± 0.034%, COMBI = 0.601 ± 0.028%, MIGLU = 0.551 ± 0.037%, HPßCD = 0.501 ± 0.035%, HPßCD1x = 0.503 ± 0.058% groups (Figure 2C). The relative weights of the male *Npc1^−/−^* mice of all groups—except for a higher value in the Sham-treated mice (0.691 ± 0.032%)—were in a comparable range (0.460–0.607%) and did not differ significantly. Thus, the relative weight of hearts in the Sham group was significantly greater compared with the None (0.532 ± 0.023%), MIGLU (0.476 ± 0.04%), HPßCD (0.527 ± 0.041%), and HPßCD1x (0.460 ± 0.052%) groups (Figure 2C).

Female mice: The heart weights of female *Npc1^+/+^* mice of all groups were not significantly different: None (0.115 ± 0.00357 g), Sham (0.120 ± 0.00684 g), COMBI (0.112 ± 0.00471 g), MIGLU (0.104 ± 0.00649 g), HPßCD (0.101 ± 0.00684 g) and HPßCD1x (0.100 ± 0.00530 g) (Figure 2B). Heart weights of female *Npc1^−/−^* mice of the None (0.0746 ± 0.00428 g) and Sham (0.0824 ± 0.00592 g) groups did not differ significantly. The weights of the COMBI group (0.105 ± 0.00548 g) were significantly higher than the values of the None and HPßCD1x groups (Figure 2B). Female *Npc1^−/−^* mice of the None, Sham, and HPßCD1x groups had significantly smaller hearts compared with the respective *Npc1^+/+^* mice (Figure 2B). Thus, COMBI, MIGLU, and HPßCD treatment normalized the heart weights of female *Npc1^−/−^* mice (Figure 2B).

In all mice of the 12 female *Npc1^−/−^* groups, the relative heart weights were in a similar range between 0.485 and 0.577% of body weight (Figure 2D).

#### 2.2.2. Lungs (Both Sides)

Left and right lungs were weighed together because of their known side-specific anatomical differences in the mouse [99,100,101].

**Figure 3:**
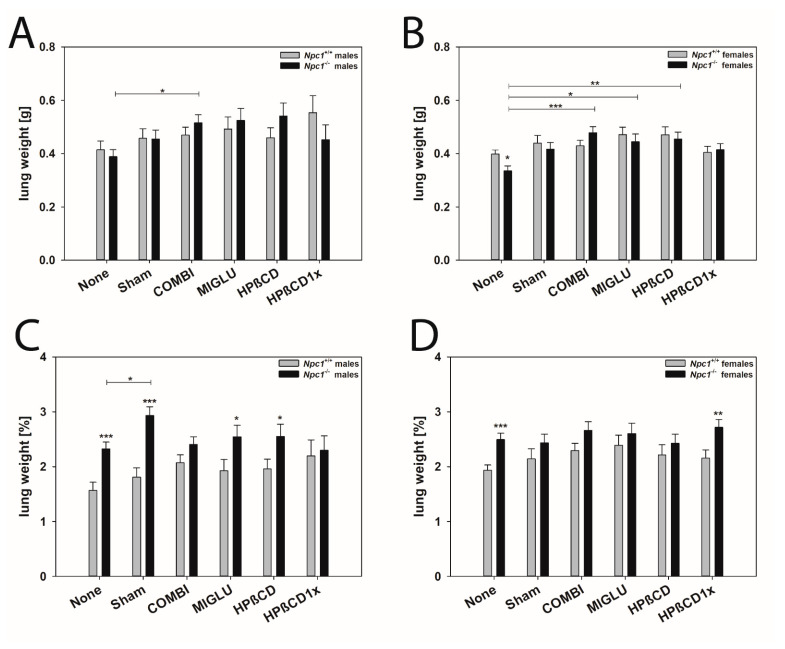
Lung weight (**A**,**B**) and relative lung weight (**C**,**D**) of *Npc1* mice. Male (**A**,**C**) and female (**B**,**D**) mice. Significant post-hoc tests are indicated by asterisks (* *p* < 0.05, ** *p* < 0.01, *** *p* < 0.001). Data are means ± SEM.

Male mice: The lung weights of male *Npc1^+/+^* mice of all six groups were in the range of 0.414 to 0.554 g and did not differ significantly between groups (Figure 3A). In male *Npc1^−/−^* mice, weights were comparable (range: 0.388 to 0.542 g). Only the lung weights of the COMBI group (0.516 ± 0.030 g) significantly surpassed the values of the None group (0.388 ± 0.026 g) (*p* = 0.028) (Figure 3A). The lung weights of the male *Npc1^+/+^* and *Npc1^−/−^* mice in the respective experimental groups did not differ significantly.

The relative lung weights of the male *Npc1^+/+^* mice of all groups were in the range of 1.75 to 2.20% and did not differ significantly between the treatment groups (Figure 3C). The same holds true for the relative lung weights of the male *Npc1^−/−^* mice, except for larger values in the Sham group (Figure 3C). The relative lung weights of the male *Npc1^−/−^* mice generally exceeded those of the respective group of *Npc1^+/+^* mice, reaching a significant level in the None, Sham, MIGLU, and HPßCD groups.

Female mice: The lung weights of female *Npc1^+/+^* mice of all six groups did not differ significantly between groups: None (0.398 ± 0.0153 g), Sham (0.439 ± 0.0289 g), COMBI (0.429 ± 0.0204 g), MIGLU (0.471 ± 0.0289 g), HPßCD (0.470 ± 0.0306 g), and HPßCD1x (0.404 ± 0.0232 g) showed no significant differences (Figure 3B). The lungs of the female *Npc1^−/−^* mice in the None (0.335 ± 0.0185 g) and Sham (0.416 ± 0.0250 g) groups did not differ significantly (Figure 3B). The weights of the COMBI (0.478 ± 0.0240 g), HPßCD (0.454 ± 0.0261 g), and MIGLU (0.444 ± 0.0289 g) groups were significantly higher compared with the values of the None group (Figure 3B). Comparison of female *Npc1^+/+^* and *Npc1^−/−^* mice revealed, with the exception of smaller weights in the *Npc1^−/−^* mice of the None group, that there were no significant differences in lung weight (Figure 3B).

The relative lung weights of all female *Npc1^+/+^* mice were in the range of 1.93 to 2.39%, without significant differences between the respective treatment groups (Figure 3D). Also, no significant intergroup differences were found in female *Npc1^−/−^* mice (Figure 3D).

Although the relative lung weights in all-female groups tentatively exceeded the respective values of the *Npc1^+/+^* mice, significantly increased relative lung weights were only found in the None and HPßCDx1 groups (Figure 3D).

### 2.3. Abdominal Organ Weights

#### 2.3.1. Liver

**Figure 4:**
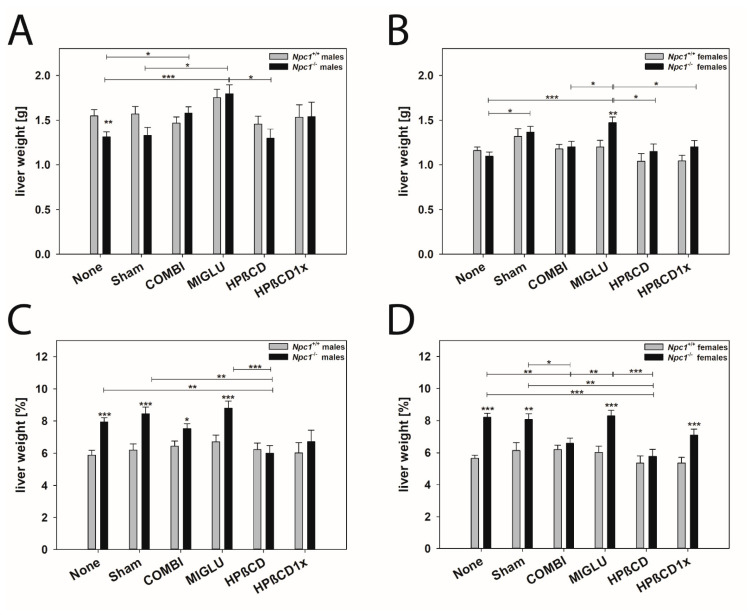
Liver weight (**A**,**B**) and relative liver weight (**C**,**D**) of *Npc1* mice. Male (**A**,**C**) and female (**B**,**D**) mice. Significant post-hoc tests are indicated by asterisks (* *p* < 0.05, ** *p* < 0.01, *** *p* < 0.001). Data are means ± SEM.

Male mice: The liver weights of the male *Npc1^+/+^* mice of groups None (1.549 ± 0.0699 g) and Sham (1.570 ± 0.0843 g) did not differ significantly (Figure 4A). The weights of the groups COMBI (1.469 ± 0.0678 g), MIGLU (1.752 ± 0.0932 g), HPßCD (1.457 ± 0.0884 g), and HPßCD1x (1.534 ± 0.140 g) also were not significantly different from the values of the groups None and Sham (Figure 4A). The liver weights of the male *Npc1^−/−^* mice in groups None (1.312 ± 0.0559 g) and Sham (1.327 ± 0.0932 g) were in the same range, as were the weights of groups HPßCD (1.296 ± 0.106 g) and HPßCD1x (1.540 ± 0.161 g) (Figure 4A). The liver weights of the None or Sham group were significantly exceeded by the COMBI (1.580 ± 0.0699 g) and the MIGLU (1.796 ± 0.0989 g) groups (Figure 4A). A comparison of the respective treatment groups revealed that only the *Npc1^−/−^* None group had significantly smaller livers than the wild-type mice (Figure 4A).

The relative liver weights of the male *Npc1^+/+^* mice of all groups were in the range of 5.88 to 6.71% without significant differences between the treatment groups (Figure 4C): None (5.88 ± 0.031%) and Sham (6.21 ± 0.38%), COMBI (6.45 ± 0.30%), MIGLU (6.71 ± 0.42%), HPßCD (6.24 ± 0.39%), and HPßCD1x (6.03 ± 0.62%) (Figure 4C). The relative liver weights of the *Npc1^−/−^* mice of groups None (7.94 ± 0.25%), Sham (8.46 ± 0.42%), and MIGLU (8.79 ± 0.44%) were significantly higher than those of the HPßCD group (6.01 ± 0.47%) (Figure 4C). The relative weights of the *Npc1^−/−^* mice of the None, Sham, COMBI, and MIGLU groups significantly exceeded the values of the respective *Npc1^+/+^* groups (Figure 4C).

Female mice: The liver weights of the female *Npc1^+/+^* mice of all groups were about 1.16 g, showing no significant differences: None (1.160 ± 0.038 g), Sham (1.316 ± 0.090 g), COMBI (1.177 ± 0.0503 g), MIGLU (1.199 ± 0.073 g), HPßCD (1.041 ± 0.083 g), and HPßCD1x (1.044 ± 0.063 g) (Figure 4B). The liver weights of the female *Npc1^−/−^* mice were highest in the MIGLU group (1.473 ± 0.063 g), significantly exceeding the groups None (1.097 ± 0.046 g), COMBI (1.202 ± 0.0503 g), HPßCD (1.148 ± 0.083 g) and HPßCD1x (1.202 ± 0.069 g) (Figure 4B). A comparison of liver weights of the respective female *Npc1^−/−^* and *Npc1^+/+^* mice groups revealed a significantly higher liver weight in the MIGLU group of the *Npc1^−/−^* mice (Figure 4B).

The relative liver weights of the female *Npc1^−/−^* mice of the groups None (8.20 ± 0.25%), Sham (8.07 ± 0.36%), and MIGLU (8.29 ± 0.34%) were significantly higher than those of the COMBI (6.58 ± 0.33%), HPßCD (5.77 ± 0.45%), and HPßCD1x (7.09 ± 0.38%) groups (Figure 4D). Moreover, the relative liver weights of the *Npc1^−/−^* mice of the None, Sham, MIGLU, and HPßCD1x groups were significantly higher compared with the respective groups of the *Npc1^+/+^* mice (Figure 4D).

#### 2.3.2. Spleen

**Figure 5:**
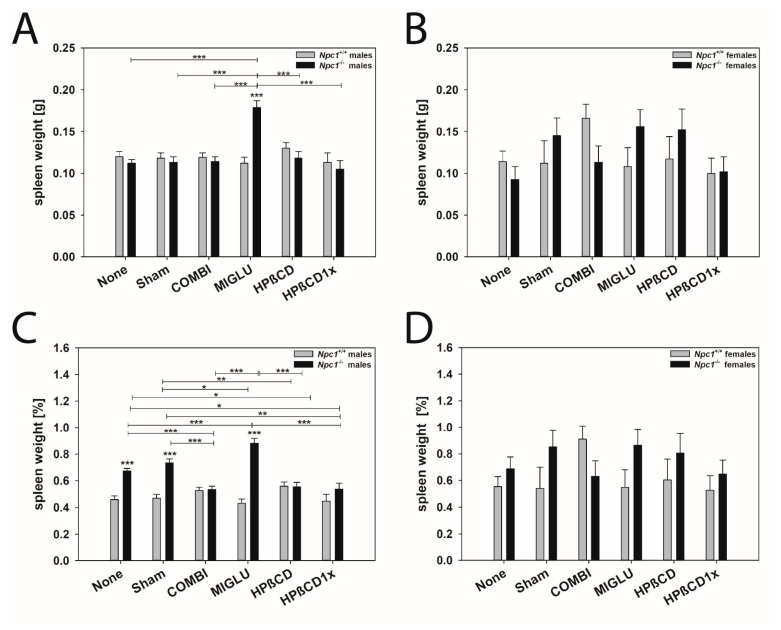
Spleen weight (**A**,**B**) and relative spleen weight (**C**,**D**) of *Npc1* mice. Male (**A**,**C**) and female (**B**,**D**) mice. Significant post-hoc tests are indicated by asterisks (* *p* < 0.05, ** *p* < 0.01, *** *p* < 0.001). Data are means ± SEM.

Male mice: The spleen weights of the male *Npc1^+/+^* mice of the groups None (0.120 ± 0.0061 g) and Sham (0.118 ± 0.0065 g) as well as of the groups COMBI (0.119 ± 0.0055 g), MIGLU (0.112 ± 0.0072 g), HPßCD (0.130 ± 0.0068 g) and HPßCD1x (0.113 ± 0.011 g) showed no significant differences (Figure 5A). Also, the spleen weights of the male *Npc1^−/−^* mice of groups None (0.112 ± 0.0044 g) and Sham (0.113 ± 0.0068 g) and those of the groups COMBI (0.114 ± 0.0057 g), HPßCD (0.118 ± 0.0080 g), and HPßCD1x (0.105 ± 0.010 g) did not reveal significant differences. Only the spleen weights of the *Npc1^−/−^* MIGLU group (0.179 ± 0.0080 g) were significantly higher than all other groups (*p* < 0.001) (Figure 5A). A comparison of the respective treatment groups revealed that only the spleens of the MIGLU-treated *Npc1^−/−^* mice had significantly heavier spleens than the wild-type mice (Figure 5A).

The relative spleen weights of the *Npc1^+/+^* mice of the groups None (0.458 ± 0.0273%) and Sham (0.469 ± 0.0295%) were in the range of all other groups: COMBI (0.526 ± 0.0248%), MIGLU (0.431 ± 0.0323%), HPßCD (0.559 ± 0.0308%) and HPßCD1x (0.447 ± 0.0510%) showed no significant differences between the groups (Figure 5C). In the male *Npc1^−/−^* mice, the relative spleen weights of the groups None (0.673 ± 0.0200%), Sham (0.734 ± 0.0308%), and MIGLU (0.881 ± 0.0361%) significantly exceeded those of the groups COMBI (0.533 ± 0.0255%), HPßCD (0.553 ± 0.0361%) and HPßCD1x (0.537 ± 0.0457%). Moreover, the relative spleen weights of the *Npc1^−/−^* mice of the None, Sham, and MIGLU groups were significantly higher (*p* < 0.001) compared with the same groups of the *Npc1^+/+^* mice (Figure 5C).

Female mice: The spleen weights of the female *Npc1^+/+^* and *Npc1^−/−^* mice of all groups did not show any significant differences, probably due to the unexpectedly high interindividual variability, only seen in this organ of female mice (Figure 5B). Likewise, the relative spleen weights of the *Npc1^+/+^* and *Npc1^−/−^* mice of all groups did not show significant differences (Figure 5D).

### 2.4. Retroperitoneal Organ Weight

#### 2.4.1. Kidney (Mean of Both Sides)

**Figure 6:**
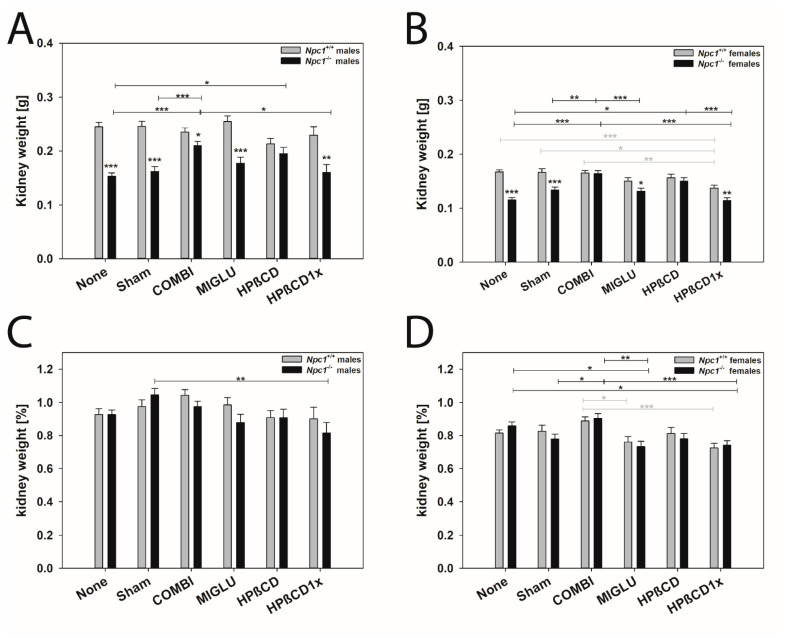
Kidney weight (**A**,**B**) and relative kidney weight (**C**,**D**) of *Npc1* mice. Male (**A**,**C**) and female (**B**,**D**) mice. Significant post-hoc tests are indicated by asterisks (* *p* < 0.05, ** *p* < 0.01, *** *p* < 0.001). Data are means ± SEM.

Male mice: The kidney weights of the male *Npc1^+/+^* mice of all groups did not show significant differences: None (0.245 ± 0.0084 g), Sham (0.246 ± 0.0094 g), COMBI (0.235 ± 0.0079 g), MIGLU (0.255 ± 0.0103 g), HPßCD (0.213 ± 0.0099 g), and HPßCD1x (0.229 ± 0.0163 g) (Figure 6A). In the male *Npc1^−/−^* mice, kidney weights of groups None (0.153 ± 0.0064 g) and Sham (0.162 ± 0.0091 g) did not differ significantly (Figure 6A). The weights of the groups MIGLU (0.177 ± 0.0115 g) and HPßCD1x (0.160 ± 0.0146 g) showed no significant differences from the values of the groups None and Sham. However, the weights of the COMBI (0.210 ± 0075 g) and HPßCD (0.195 ± 0.0115 g) groups significantly exceeded the values of the None group (Figure 6A). With the exception of the HPßCD group, the kidney weights of all male *Npc1^−/−^* mice groups were significantly lower than those of the comparable *Npc1^+/+^* groups (Figure 6A).

The relative kidney weights of the male *Npc1^+/+^* mice in all groups did not differ significantly: None (0.927 ± 0.0364%), Sham (0.975 ± 0.0407%), COMBI (1.043 ± 0.0342%), MIGLU (0.985 ± 0.0446%), HPßCD (0.909 ± 0.0425%), and HPßCD1x (0.901 ± 0.0704%) (Figure 6C). The relative kidney weights of the male *Npc1^−/−^* mice of the groups None (0.927 ± 0.0276%), Sham (1.045 ± 0.0391%), COMBI (0.975 ± 0.0323%), MIGLU (0.879 ± 0.0498%), and HPßCD (0.909 ± 0.0498%) groups did not show significant differences (Figure 6C). Only the relative kidney weights of the Sham group significantly exceeded those of the HPßCD1x (0.816 ± 0.0630%) group (Figure 6C). There were no significant differences in the relative kidney weights of *Npc1^+/+^* and *Npc1^−/−^* mice when comparing the respective experimental groups(Figure 6C).

Female mice: There were considerable differences in the kidney weights of the female *Npc1^+/+^* mice between different groups. Kidneys of the HPßCD1x group (0.137 ± 0.0053 g) were significantly smaller compared with the None (0.167 ± 0.0036 g), Sham (0.166 ± 0.0068 g), and COMBI (0.165 ± 0.0045 g) groups (Figure 6B), but did not differ significantly from the MIGLU (0.150 ± 0.0064 g) and HPßCD (0.156 ± 0.0068 g) groups (Figure 6B). The kidney weights of the female *Npc1^−/−^* mice in the groups None (0.115 ± 0.0045 g) and Sham (0.133 ± 0.0059 g) were in the range of the MIGLU (0.131 ± 0.0059 g) and HPßCD1x (0.114 ± 0.0051 g) groups (Figure 6B). With the exception of the COMBI and HPßCD groups, the kidney weights of the other female *Npc1^−/−^* mouse groups were significantly lower than those of the comparable *Npc1^+/+^* groups (Figure 6A).

In the *Npc1^+/+^* mice, the relative kidney weights of the groups None (0.815 ± 0.0194%) and Sham (0.827 ± 0.0366%) did not differ significantly (Figure 6D) from the other experimental groups. Only the relative weights of the COMBI group (0.889 ± 0.0245%) exceeded the values of the MIGLU (0.760 ± 0.0347%) and HPßCD1x (0.724 ± 0.0283%) groups significantly (Figure 6D). The relative kidney weights of the *Npc1^−/−^* mice of the groups None (0.859 ± 0.0245%) and Sham (0.778 ± 0.0317%) were not significantly different. However, the values found in the MIGLU (0.733 ± 0.0317%) and HPßCD1x (0.741 ± 0.0274%) groups were significantly smaller (Figure 6D). The comparison of the respective data between *Npc1^+/+^* and *Npc1^−/−^* mice of the same experimental groups showed no significant differences (Figure 6D).

#### 2.4.2. Adrenal Gland (Mean of Both Sides)

**Figure 7:**
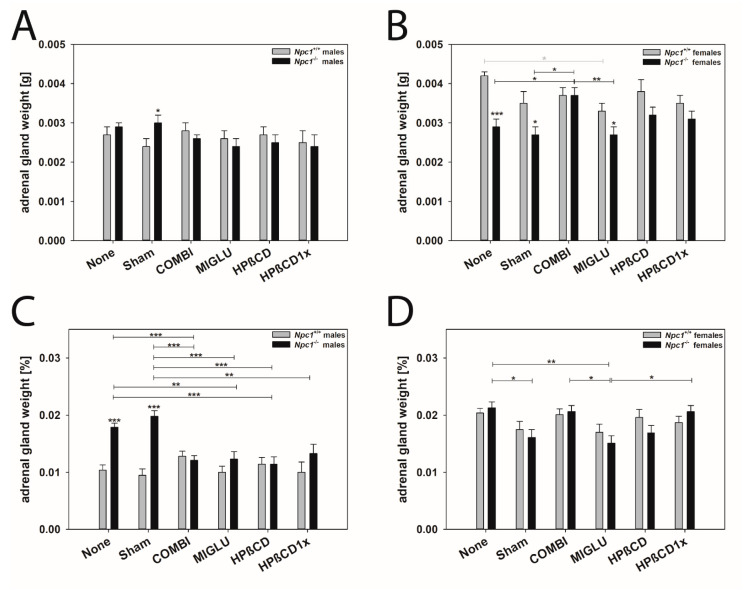
Adrenal gland weight (**A**,**B**) and relative adrenal gland weight (**C**,**D**) of *Npc1* mice. Male (**A**,**C**) and female (**B**,**D**) mice. Significant post-hoc tests are indicated by asterisks (* *p* < 0.05, ** *p* < 0.01, *** *p* < 0.001). Data are means ± SEM.

Male mice: The adrenal gland weights of the male *Npc1^+/+^* mice of groups None (0.00273 ± 0.00016 g) and Sham (0.00237 ± 0.00019 g) did not differ significantly from the values of the other groups: COMBI (0.00280 ± 0.00016 g), MIGLU (0.00259 ± 0.00019 g), HPßCD (0.00273 ± 0.00020 g), and HPßCD1x (0.00251 ± 0.00030 g) (Figure 7A). Also, in the male *Npc1^−/−^* mouse groups the adrenal gland weight was in a similar range: None (0.00292 ± 0.00012 g), Sham (0.00303 ± 0.00017 g), COMBI (0.00259 ± 0.00014 g), MIGLU (0.00244 ± 0.00022 g), HPßCD (0.00246 ± 0.00023 g), and HPßCD1x (0.00244 ± 0.00027 g) (Figure 7A). When comparing wild-types and mutants, only the None *Npc1^−/−^* mice had larger adrenal glands than the corresponding wild-types.

The relative adrenal gland weights of the *Npc1^+/+^* mice of the groups None (0.0104 ± 0.0009%) and Sham (0.0095 ± 0.0011%) did not differ significantly from values of the COMBI (0.0128 ± 0.0009%), MIGLU (0.0100 ± 0.0011%), HPßCD (0.0114 ± 0.0012%), and HPßCD1x (0.0100 ± 0.0018%) groups (Figure 7C).

In the *Npc1^−/−^* mice of the groups None (0.0179 ± 0.0007%) and Sham (0.0198 ± 0.0010%), the relative adrenal gland weights were significantly higher compared with the MIGLU (0.0123 ± 0.0013%), HPßCD (0.0114 ± 0.0013%), COMBI (0.0121 ± 0.0008%), and HPßCD1x (0.0133 ± 0.0016%) groups. When comparing wild-types and mutants, only the None and Sham *Npc1^−/−^* mice had highly significantly larger adrenal glands than the corresponding wild-types (Figure 7C).

Female mice: The adrenal gland weights of the female *Npc1^+/+^* mice of groups None (0.00419 ± 0.00013 g), Sham (0.00354 ± 0.00025 g), COMBI (0.00373 ± 0.00017 g), HPßCD (0.00376 ± 0.00025 g), and HPßCD1x (0.00352 ± 0.00019 g) did not differ significantly (Figure 7B). The weights of the MIGLU group (0.00332 ± 0.00024 g) were significantly lower than the values of the None group (*p* = 0.024) of the female *Npc1^+/+^* mice (Figure 7B). The adrenal gland weights of the female *Npc1^−/−^* mice in the None (0.00286 ± 0.00017 g) and Sham (0.00272 ± 0.00024 g) groups did not differ significantly (Figure 7B). The weights of the groups MIGLU (0.00267 ± 0.00022 g), HPßCD (0.00320 ± 0.00023 g), and HPßCD1x (0.00313 ± 0.00019 g) were in the same range and did not show significant differences from the values of groups None and Sham (Figure 7B). Only the weights of the COMBI group (0.00374 ± 0.00019 g) were significantly higher compared with the values of the MIGLU, None, and Sham groups (*p* = 0.005 and *p* = 0.012 and *p* = 0.015) (Figure 7B).

The relative adrenal gland weights of the female *Npc1^+/+^* mice of all groups were in the range of 0.0170–0.0204%, showing no significant differences: None (0.0204 ± 0.0008%), Sham (0.0175 ± 0.0014%), COMBI (0.0201 ± 0.0010%), MIGLU (0.0170 ± 0.0014%), HPßCD (0.0196 ± 0.0014%), and HPßCD1x (0.0187 ± 0.0011%) (Figure 7D). The respective data of the *Npc1^−/−^* mice, however, differed. The groups None (0.0213 ± 0.0010%), COMBI (0.0206 ± 0.0011%), and HPßCD1x (0.0206 ± 0.0011%) groups significantly exceeded the MIGLU (0.0151 ± 0.0013%) mice (Figure 7D). A comparison of wild-types and mutants revealed no significant differences with respect to their relative adrenal gland weights (Figure 7D).

### 2.5. Pelvic Organ Weight

#### Bladder

**Figure 8:**
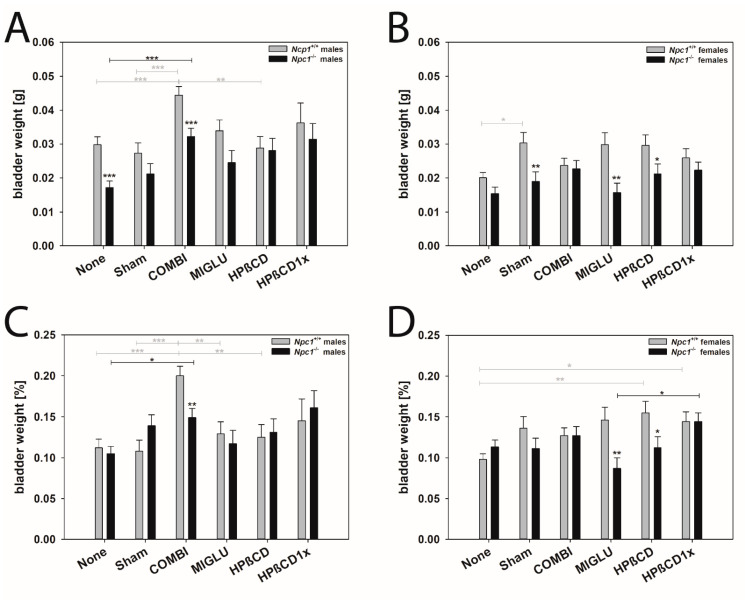
Bladder weight (**A**,**B**) and relative bladder weight (**C**,**D**) of *Npc1* mice. Male (**A**,**C**) and female (**B**,**D**) mice. Significant post-hoc tests are indicated by asterisks (* *p* < 0.05, ** *p* < 0.01, *** *p* < 0.001). Data are means ± SEM.

Male mice: The bladder weights of the male *Npc1^+/+^* mice of groups None (0.0298 ± 0.0023 g), Sham (0.0273 ± 0.0030 g), MIGLU (0.0339 ± 0.0032 g), HPßCD (0.0288 ± 0.0034 g), and HPßCD1x (0.0362 ± 0.0059 g) showed no significant differences (Figure 8A). However, the weights of the COMBI group (0.0444 ± 0.0026 g) were significantly higher compared with the values of the Sham, None, and HPßCD groups (Figure 8A). Except for the male *Npc1^−/−^* mice in group COMBI (0.0322 ± 0.0024 g), the bladder weights of all others were in a comparable range: None (0.0172 ± 0.0019 g), Sham (0.0212 ± 0.0030 g), MIGLU (0.0245 ± 0.0036 g), HPßCD (0.0281 ± 0.0036 g), and HPßCD1x (0.0314 ± 0.0046 g) showed no significant differences (Figure 8A). The weights of the COMBI group (0.0322 ± 0.0024 g) were significantly higher than the values of the None group (*p* < 0.001) (Figure 8A). In the male *Npc1^−/−^* mice, the bladder weights of the None and COMBI groups were significantly lower compared with the appropriative groups of the *Npc1^+/+^* mice (each *p* < 0.001).

The relative bladder weights of the male *Npc1^+/+^* mice of groups None (0.112 ± 0.011%), Sham (0.108 ± 0.013%), MIGLU (0.129 ± 0.015%), HPßCD (0.125 ± 0.016%), and HPßCD1x (0.145 ± 0.027%) were in a comparable range (Figure 8C). However, the values of the COMBI group (0.200 ± 0.012%) were significantly higher compared with the values of the None group (Figure 8C). Except for the male *Npc1^−/−^* mice of the COMBI group (0.149 ± 0.011%), the relative bladder weights of all others were in a comparable range: None (0.105 ± 0.009%), Sham (0.139 ± 0.013%), MIGLU (0.117 ± 0.016%), HPßCD (0.131± 0.016%), and HPßCD1x (0.161 ± 0.021%) and showed no significant differences (Figure 8C). The weights of the COMBI group (0.200 ± 0.012 g) were significantly higher than the values of the None group (*p* = 0.002) (Figure 8C). Only the relative bladder weight of the *Npc1^−/−^* mice of the COMBI group was significantly lower than that of the same groups of *Npc1^+/+^* mice (Figure 8C).

Female mice: The bladder weights of the female *Npc1^+/+^* mice of all groups—except for a higher weight in the Sham group (0.0303 ± 0.0031 g)—were in a comparable range: None (0.0201 ± 0.0015 g), COMBI (0.0237 ± 0.0021 g), MIGLU (0.0298 ± 0.0035 g), HPßCD (0.0296 ± 0.0031 g), and HPßCD1x (0.0259 ± 0.0027 g) (Figure 8B). The bladder weights of the female *Npc1^−/−^* mice showed no significant differences between the groups: None (0.0154 ± 0.0019 g), Sham (0.0190 ± 0.0028 g), COMBI (0.0227 ± 0.0024 g), MIGLU (0.0157 ± 0.0028 g), HPßCD (0.0212 ± 0.0029 g), and HPßCD1x (0.0223 ± 0.0024 g) (Figure 8B). In the groups Sham, MIGLU, and HPßCD, the bladder weights of the *Npc1^−/−^* groups were significantly smaller compared with the corresponding groups of wild-type mice (Figure 8B).

The relative weights of the *Npc1^+/+^* mice of the None group (0.098 ± 0.007%) fell significantly below the values of groups HPßCD (0.155 ± 0.014%) and HPßCD1x (0.144 ± 0.012%) (Figure 8D). The relative weights of groups Sham (0.136 ± 0.014 g), COMBI (0.127 ± 0.010 g), and MIGLU (0.146 ± 0.016 g) did not show significant differences from the values the group None (Figure 8D). The relative bladder weight of the *Npc1^−/−^* mice of the MIGLU and HPßCD groups was significantly lower than in the respective wild-type groups (Figure 8D).

### 2.6. Genital Organ Weight

#### 2.6.1. Testis plus Epididymis (Mean of Both Sides)

**Figure 9:**
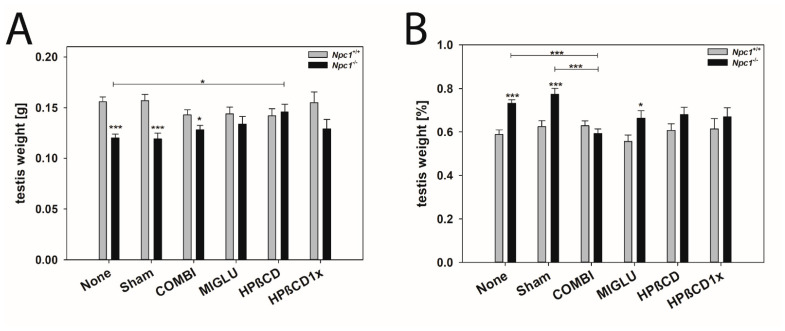
Testis weight (**A**) and relative testis weight (**B**) of *Npc1* mice. Significant post-hoc tests are indicated by asterisks (* *p* < 0.05, *** *p* < 0.001). Data are means ± SEM.

The testis weights of the *Npc1^+/+^* mice in all groups were in a comparable range of values: None (0.156 ± 0.0048 g), Sham (0.157 ± 0.0061 g), COMBI (0.143 ± 0.0051 g), MIGLU (0.144 ± 0.0066 g), HPßCD (0.142 ± 0.0070 g), and HPßCD1x (0.155 ± 0.011 g) (Figure 9A). In *Npc1^−/−^* mice, significantly different values were only found between group None (0.120 ± 0.0038 g) and the HPßCD group (0.146 ± 0.0074 g) (Figure 9A). The testis weights of the *Npc1^−/−^* mice of the None (0.120 ± 0.0038 g), Sham (0.119 ± 0.0058 g), and COMBI (0.128 ± 0.0048 g) groups were significantly lower compared with the corresponding groups of the *Npc1^+/+^* mice (Figure 9A).

The relative testis weights in all *Npc1^+/+^* mice groups were not significantly different: None (0.588 ± 0.022%), Sham (0.625 ± 0.027%), COMBI (0.629 ± 0.023%), MIGLU (0.556 ± 0.030%), HPßCD (0.607 ± 0.032%), and HPßCD1x (0.615 ± 0.047%) (Figure 9B). The relative testis weights of the *Npc1^−/−^* mice of the groups None (0.732 ± 0.017%) and Sham (0.774 ± 0.026%) significantly exceeded the values of the COMBI-treated ones (Figure 9B). The relative testis weights of the *Npc1^−/−^* mice of the None, Sham, and MIGLU groups were significantly higher compared with the corresponding groups of the *Npc1^+/+^* mice (Figure 9B).

#### 2.6.2. Vesicular Gland and Scent Gland (Mean of Both Sides)

**Figure 10:**
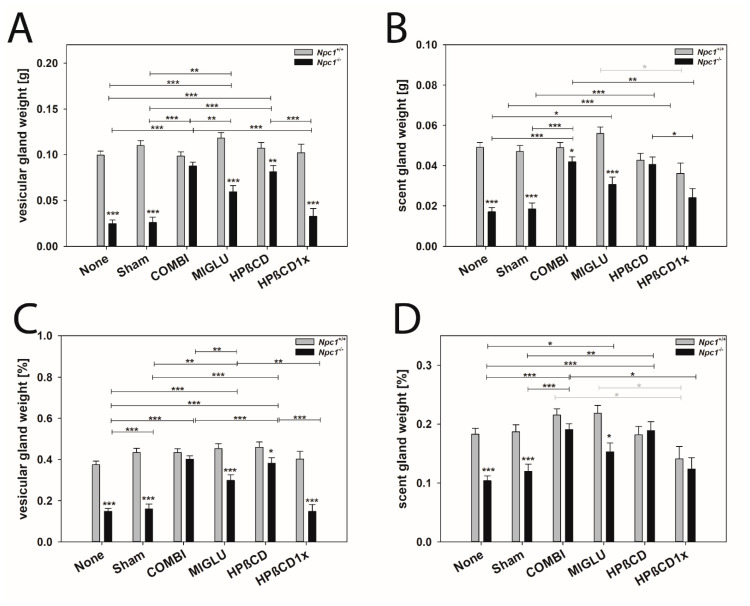
Vesicular gland weight (**A**) and scent gland weight (**B**), relative vesicular gland weight (**C**), and relative scent gland weight (**D**) of *Npc1* mice. Significant post-hoc tests are indicated by asterisks (* *p* < 0.05, ** *p* < 0.01, *** *p* < 0.001). Data are means ± SEM.

The weights of the vesicular glands of the Npc1*^+/+^* mice of all groups were comparable: None (0.0995 ± 0.0043 g), Sham (0.110 ± 0.0054 g), COMBI (0.0985 ± 0.0046 g), MIGLU (0.118 ± 0.0060 g), HPßCD (0.107 ± 0.0063 g), and HPßCD1x (0.102 ± 0.0094 g) (Figure 10A). The weights of the vesicular glands of the *Npc1^−/−^* mice, however, differed: groups None (0.0248 ± 0.0038 g) and Sham (0.0259 ± 0.0060 g) had significantly lower weights compared with COMBI (0.0875 ± 0.0043 g), MIGLU (0.0875 ± 0.00431 g), and HPßCD groups (0.0813 ± 0.0067 g), but had values at the same low level as the HPßCD1x group (0.0328 ± 0.0084 g) (Figure 10A). Comparing wild-type and mutant mice, the weights of the vesicular glands of the *Npc1^−/−^* groups None, Sham, MIGLU, HPßCD, and HPßCD1x were significantly smaller compared with the corresponding *Npc1^+/+^* mice (Figure 10A).

Nearly identical differences between experimental groups were observed in relative vesicular gland weights of *Npc1^+/+^* and *Npc1^−/−^* mice (Figure 10C).

The scent gland weights of the *Npc1^+/+^* mice of groups None (0.0491 ± 0.0024 g) and Sham (0.0470 ± 0.0030 g) did not differ significantly from the weights of the groups COMBI (0.0489 ± 0.0026 g), MIGLU (0.0559 ± 0.0032 g), HPßCD (0.0427 ± 0.0034 g) and HPßCD1x (0.0361 ± 0.0051 g). However, the scent glands of the MIGLU group were significantly heavier than those of the HPßCD1x group (*p* = 0.021) (Figure 10B). *Npc1^−/−^* mice of groups None (0.0171 ± 0.0021 g) and Sham (0.0185 ± 0.0030 g) had similar scent gland weights (Figure 10B). With the exception of the HPßCD1x group (0.0241 ± 0.0046 g), the COMBI (0.0419 ± 0.0024 g), MIGLU (0.0307 ± 0.0036 g), and HPßCD (0.04106 ± 0.0036 g) groups had significantly higher scent gland weights than the None or Sham groups (Figure 10B). The weights of the scent glands of *Npc1^−/−^* mice in the None, Sham, COMBI, and MIGLU groups were significantly lower than those in the same groups of *Npc1^+/+^* mice (Figure 10B).

The relative scent gland weights of the *Npc1^+/+^* mice of the groups None (0.183 ± 0.010%) and Sham (0.187 ± 0.012%) did not differ significantly from the respective values of the COMBI (0.216 ± 0.011%), MIGLU (0.219 ± 0.013%), HPßCD (0.182 ± 0.014%) and HPßCD1x (0.141 ± 0.021%) groups (Figure 10D). *Npc1^−/−^* mice of groups None (0.104 ± 0.008%) and Sham (0.120 ± 0.012%) had similar relative scent gland weights (Figure 10D). With the exception of the HPßCD1x group (0.124 ± 0.019%), the COMBI (0.191 ± 0.010%), MIGLU (0.153 ± 0.015%), and HPßCD (0.189 ± 0.015%) groups had significantly higher relative scent gland weights than the None or Sham groups (Figure 10B). The relative weights of the scent glands of *Npc1^−/−^* mice in the None, Sham, and MIGLU groups were significantly lower than those in the same groups of *Npc1^+/+^* mice (Figure 10D).

#### 2.6.3. Ovary (Mean of Both Sides)

**Figure 11:**
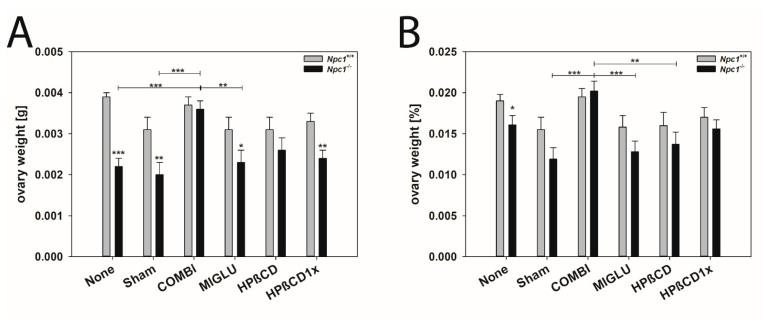
Ovary weight (**A**) and relative ovary weight (**B**) of *Npc1* mice. Significant post-hoc tests are indicated by asterisks (* *p* < 0.05, ** *p* < 0.01, *** *p* < 0.001). Data are means ± SEM.

The ovarian weights of *Npc1^+/+^* mice in the None (0.00389 ± 0.00015 g), Sham (0.00312 ± 0.00028 g), COMBI (0.00367 ± 0.00019 g), MIGLU (0.00312 ± 0.00026 g), HPßCD (0.00312 ± 0.00029 g), and HPßCD1x (0.00330 ± 0.00023 g) were all in a comparable range with no significant differences between their values (Figure 11A). The ovarian weights of the *Npc1^−/−^* mice of groups None (0.00218 ± 0.00021 g), Sham (0.00203 ± 0.00026 g), MIGLU (0.00234 ± 0.00025 g), HPßCD (0.00261 ± 0.00028 g), and HPßCD1x (0.00235 ± 0.00021 g) were comparably low without significant differences (Figure 11A). Only the COMBI-treated mice (0.00363 ± 0.00022 g) had statistically bigger ovaries compared to the MIGLU, None, and Sham groups. The ovaries of the *Npc1^−/−^* groups None, Sham, MIGLU, and HPßCD1x were significantly smaller compared with the *Npc1^+/+^* mice (Figure 11A).

The relative ovary weights of the groups of *Npc1^+/+^* mice were in the same range: None (0.0190 ± 0.0008%), Sham (0.0155 ± 0.0015%), COMBI (0.0195 ± 0.0010%), MIGLU (0.0158 ± 0.0014%), HPßCD (0.0160 ± 0.0016%), and HPßCD1x (0.0170 ± 0.0012%) were without significant differences (Figure 11B). The respective values in *Npc1^−/−^* mice revealed that the relative weights in the COMBI-treated group were significantly higher than in the Sham (0.0119 ± 0.0014%) and MIGLU (0.0128 ± 0.0013%) and HPßCD (0.0137 ± 0.0015g) groups (Figure 11B). Only the *Npc1^−/−^* mice of the None group had a significantly lower relative ovary weight than those of the same *Npc1^+/+^* mice (Figure 11B).

#### 2.6.4. Uterus

**Figure 12:**
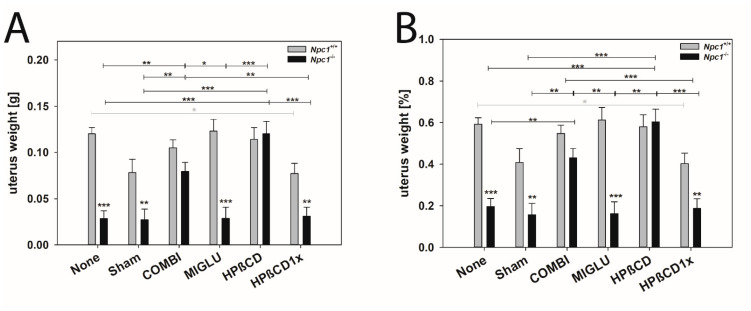
Uterus weight (**A**) and relative uterus weight (**B**) of *Npc1* mice. Significant post-hoc tests are indicated by asterisks (* *p* < 0.05, ** *p* < 0.01, *** *p* < 0.001). Data are means ± SEM.

The uterus weights of the *Npc1^+/+^* mice in the groups None (0.120 ± 0.007 g), Sham (0.0782 ± 0.014 g), COMBI (0.105 ± 0.0085 g), MIGLU (0.123 ± 0.013 g), and HPßCD (0.114 ± 0.013 g) showed no significant differences (Figure 12A). Only the weights of the HPßCD1x group (0.077 ± 0.011 g) were significantly lower compared with the None group (*p* = 0.016) (Figure 12A). The uterus weights of the *Npc1^−/−^* mice of the None (0.029 ± 0.008 g), Sham (0.027 ± 0.012 g), MIGLU (0.029 ± 0.012 g), and HPßCD1x (0.031 ± 0.010 g) groups showed no significant differences. However, the uteri of the COMBI (0.080 ± 0.010 g) and HPßCD (0.120 ± 0.013 g) groups were significantly larger than those of the None or Sham groups (Figure 12A). The weights of the *Npc1^−/−^* groups None, Sham, MIGLU, and HPßCD1x groups were significantly lower than those of the respective groups of *Npc1^+/+^* mice (Figure 12A). Nearly identical differences between experimental groups were observed in the relative uterine weights of *Npc1^+/+^* and *Npc1^−/−^* mice (Figure 12B).

### 2.7. Stomach Volume

**Figure 13:**
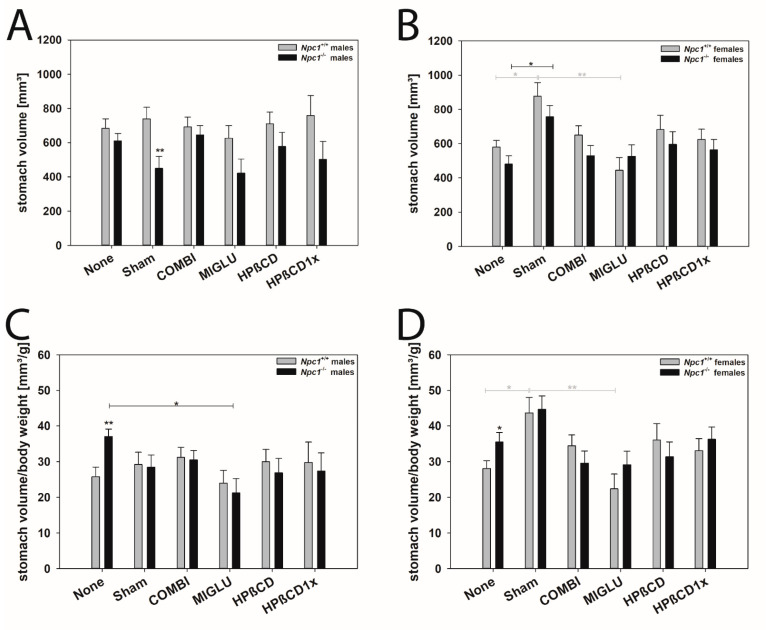
Stomach volume (**A**,**B**) and relative stomach volume (**C**,**D**) of *Npc1* mice. Male (**A**,**C**) and female (**B**,**D**) mice. Significant post-hoc tests are indicated by asterisks (* *p* < 0.05, ** *p* < 0.01). Data are means ± SEM.

Male mice: The stomach volumes of the *Npc1^+/+^* mice of all experimental groups were in the range of 625.32 to 759.93 mm^3^: None (683.15 ± 54.89 mm^3^), Sham (738.22 ± 70.21 mm^3^), COMBI (691.88 ± 58.22 mm^3^), MIGLU (625.32 ± 73.64 mm^3^), HPßCD (709.65 ± 70.21 mm^3^), and HPßCD1x (759.93 ± 116.44 mm^3^) showing no significant differences (Figure 13A). Also, the stomach volumes of the *Npc1^−/−^* mice were in the range of 421.76–645.48 mm^3^ without significant intergroup differences: None (609.81 ± 43.24 mm^3^), Sham (449.05 ± 70.21 mm^3^), COMBI (645.48 ± 53.43 mm^3^), MIGLU (421.76 ± 82.33 mm^3^), HPßCD (577.48 ± 82.33 mm^3^), and HPßCD1x (501.89 ± 104.14 mm^3^) (Figure 13A). Comparing the levels of *Npc1^+/+^* and *Npc1^−/−^* mice, Sham-treated wild-types had significantly larger stomachs than mutant mice (Figure 13A).

The relative stomach volumes (volume per body weight) of the *Npc1^+/+^* mice were similar in all groups: None (25.79 ± 2.69 mm^3^/g), Sham (29.19 ± 3.44 mm^3^/g) COMBI (31.18 ± 2.85 mm^3^/g), MIGLU (23.96 ± 3.61 mm^3^/g), HPßCD (29.99 ± 3.44 mm^3^/g), and HPßCD1x (29.79 ± 5.71 mm^3^/g) (Figure 13C). Rather similar values of relative stomach weight were also found in *Npc1^−/−^* mice: Sham (28.45 ± 3.44 mm^3^/g), COMBI (30.50 ± 2.62 mm^3^/g), HPßCD (26.85 ± 4.04 mm^3^/g), and HPßCD1x 27.38 ± 5.10 mm^3^/g) (Figure 13C). In *Npc1^−/−^* mice, the largest relative gastric volumes were found in the None (37.01 ± 2.12 mm^3^/g) group, the smallest in the MIGLU-treated group (21.23 ± 4.04 mm^3^/g) (Figure 13C). Comparing the respective levels of *Npc1^+/+^* and *Npc1^−/−^* mice, the None group of wild-types had significantly larger stomachs than the respective mutant mice (Figure 13C).

Female mice: The stomach volumes of the Sham *Npc1^+/+^* group (878.38 ± 77.86 mm^3^) significantly exceeded those of the None (579.59 ± 38.40 mm^3^) and MIGLU group (444.18 ± 73.86 mm^3^) (Figure 13B). The volumes of the COMBI (649.69 ± 53.58 mm^3^), HPßCD (681.78 ± 82.59 mm^3^), and HPßCD1x (623.99 ± 60.31 mm^3^) groups did not show significant differences from the values of the None group (Figure 13B). With the exception of the Sham-treated group (756.32 ± 67,43 mm^3^), whose gastric volume was significantly greater than that of the *Npc1^−/−^* None group (480.53 ± 47.68 mm^3^), all other volumes were within the range of the None group: COMBI (528.97 ± 60.31 mm^3^), MIGLU (525.24 ± 67.43 mm^3^), HPßCD (595.13 ± 73.86 mm^3^) and HPßCD1x (563.64 ± 60.31 mm^3^) showed no significant differences from the values of the None and Sham groups (Figure 13B). No significant differences were found when the respective groups of Npc1^−/−^ and Npc1^+/+^ mice were compared (Figure 13B).

The relative stomach volumes of the female Npc1^+/+^ mice of group Sham (43.67 ± 4.37 mm^3^/g) were significantly greater than those of the None (28.09 ± 2.15 mm^3^/g) (*p* = 0.010) and of the MIGLU groups (22.35 ± 4.14 mm^3^/g) (*p* = 0.001) (Figure 13D). The volumes of the groups COMBI (34.47 ± 3.01 mm^3^/g), HPßCD (36.06 ± 4.63 mm^3^/g), and HPßCD1x (33.09 ± 3.38 mm^3^/g) showed no significant differences to the values of the groups None and Sham (Figure 13D). The relative stomach volumes of the Npc1^−/−^ mice in all groups did not differ significantly: None (35.50 ± 2.67 mm^3^/g), Sham (44.66 ± 3.78 mm^3^/g), COMBI (29.63 ± 3.38 mm^3^/g), MIGLU (29.16 ± 3.78 mm^3^/g), HPßCD (31.38 ± 4.14 mm^3^/g), and HPßCD1x (36.29 ± 3.38 mm^3^/g). Comparing the levels of Npc1^+/+^ and Npc1^−/−^ mice, the None group of wild-types had significantly larger stomachs than the respective mutant mice (Figure 13D).

### 2.8. Femur Length

**Figure 14:**
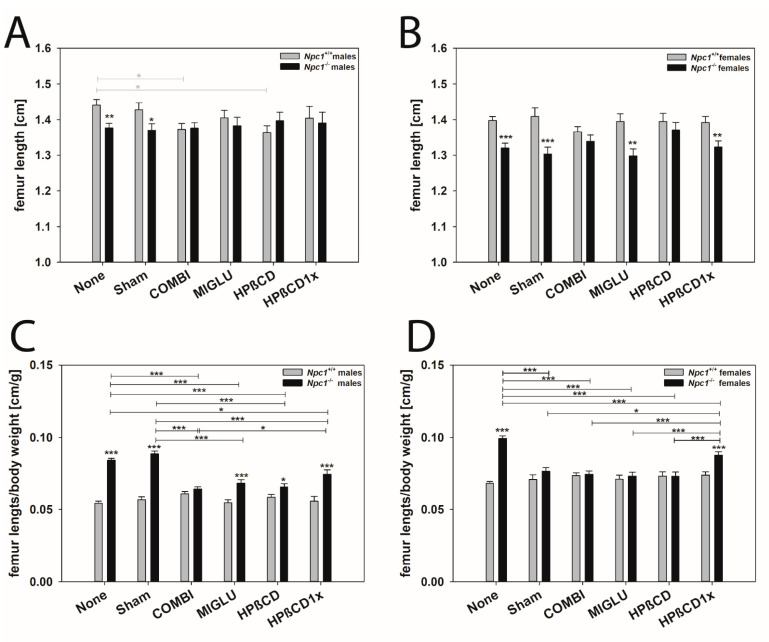
Femur length (**A**,**B**) and relative femur length (**C**,**D**) of *Npc1* mice. Male (**A**,**C**) and female (**B**,**D**) mice. Significant post-hoc tests are indicated by asterisks (* *p* < 0.05, ** *p* < 0.01, *** *p* < 0.001). Data are means ± SEM.

Male mice: The femur lengths of the *Npc1^+/+^* mice of the None (1.441 ± 0.016 cm) and Sham (1.428 ± 0.019520 cm) groups did not differ significantly (Figure 14A). The lengths of the groups MIGLU (1.405 ± 0.021 cm) and HPßCD1x (1.404 ± 0.034 cm) showed no significant differences from the values of the None and Sham groups. However, treatment with COMBI (1.373 ± 0.016 cm) or HPßCD (1.363 ± 0.020 cm) resulted in significantly shorter femurs (Figure 14A). The femurs of *Npc1^−/−^* mice in all groups were comparable: None (1.377 ± 0.013 cm), Sham (1.370 ± 0.019 cm), COMBI (1.376 ± 0.016 cm), MIGLU (1.383 ± 0.024 cm), HPßCD (1.397 ± 0.024 cm), and HPßCD1x (1.391 ± 0.030 cm) showed no significant differences. The femur lengths of the *Npc1^−/−^* None and Sham groups were significantly shorter compared to the respective wild-types (Figure 14A).

The relative femur lengths of the *Npc1^+/+^* mice in groups None (0.0543 ± 0.0015 cm/g), Sham (0.0568 ± 0.0019 cm/g), COMBI (0.0608 ± 0.0016 cm/g), MIGLU (0.0546 ± 0.0021 cm/g), HPßCD (0.0584 ± 0.0020 cm/g), and HPßCD1x (0.0557 ± 0.0033 cm/g) groups were in a comparable range showing no significant differences (Figure 14C). The relative femur lengths of the *Npc1^−/−^* mice in groups None (0.0841 ± 0.0013 cm/g) and Sham (0.0886 ± 0.0018 cm/g) did not differ but significantly exceeded the values of the COMBI (0.0641 ± 0.0015 cm/g), MIGLU (0.0683 ± 0.0024 cm/g), HPßCD (0.0655 ± 0.0024 cm/g) and HPßCD1x (0.0744 ± 0.0030 cm/g) groups (Figure 14C). The relative femur lengths of the *Npc1^−/−^* None, Sham, MIGLU, HPßCD, and HPßCD1x groups were significantly greater than in the corresponding groups of wild-type mice (Figure 14C).

Female mice: The femurs of the *Npc1^+/+^* mice of all groups had a comparable length: None (1.398 ± 0.011 cm), Sham (1.409 ± 0.024 cm), COMBI (1.366 ± 0.015 cm), MIGLU (1.395 ± 0.021 cm), HPßCD (1.395 ± 0.023 cm), and HPßCD1x (1.392 ± 0.017 cm) (Figure 14B). Likewise, in the *Npc1^−/−^* mice, all groups had similar femur lengths: None (1.320 ± 0.014 cm), Sham (1.303 ± 0.020 cm), COMBI (1.339 ± 0.017 cm), MIGLU (1.298 ± 0.020 cm), HPßCD (1.371 ± 0.021 cm), and HPßCD1x (1.323 ± 0.017 cm) (Figure 14B). The femur lengths of the *Npc1^−/−^* mice of groups None, Sham, MIGLU, and HPßCD1x were significantly smaller compared with the same groups of the *Npc1^+/+^* mice (Figure 14B).

The relative femur lengths of the *Npc1^+/+^* mice of all groups None (0.0682 ± 0.0014 cm/g), Sham (0.0708 ± 0.0032 cm/g), COMBI (0.0734 ± 0.0020 cm/g), MIGLU (0.0709 ± 0.0029 cm/g), HPßCD (0.0731 ± 0.0030 cm/g) and HPßCD1x (0.0738 ± 0.0023 cm/g) showed no significant differences (Figure 14D). In the *Npc1^−/−^* mice, the relative femur lengths of the None group (0.0994 ± 0.0018 cm/g) were significantly greater than in the Sham (0.0765 ± 0.0026 cm/g), COMBI (0.0744 ± 0.0023 cm/g), MIGLU (0.0731 ± 0.0026 cm/g), HPßCD (0.0730 ± 0.0029 cm/g), and HPßCD1x (0.0876 ± 0.0023 cm/g) groups (Figure 14D). Moreover, the *Npc1^−/−^* mice of the HPßCD1x group had significantly greater relative femur lengths compared with the Sham, COMBI, MIGLU, and HPßCD groups (Figure 14D). The femur lengths of the *Npc1^−/−^* mice of the None and HPßCD1x groups were significantly larger compared with the similar group of the *Npc1^+/+^* mice (Figure 14D).

## 3. Discussion

The organ weights of 175 *Npc1^−/−^* and 176 *Npc1^+/+^* mice are compared and discussed first with respect to gender and second with respect to the potential benefit of the therapeutic regimens used.

Systematical studies dealing with gender-specific organ weights of *Npc1^+/+^* and *Npc1^−/−^* mice are rare. With the exception of Xie et al. [102], who found that in both *Npc1^+/+^* and *Npc1^−/−^* mice, females had lower liver weights than males, all other studies with weight data, to our knowledge, examined gender-mixed groups in a different number of organs.

In addition to the brain [4,28,37,74,84,95,103,104,105,106,107,108,109], quantitative measurements of the liver [28,54,63,68,79,80,84,91,94,95,96,97,98,102,103,105,106,107,108,109,110,111,112], spleen [28,68,79,80,95,103,105,106,107,112], lung [28,68,95,103,106,113,114], heart [111], kidney [68,80,95,103], adrenal gland [95], small intestine [4,95,106,115], stomach [95], and gall bladder [38] were reported in decreasing frequency (Supplementary Materials Table S1). However, most studies described relative organ weights [28,54,63,68,80,84,97,103,107,111,112,114,116], and only a few studies reported absolute organ weights [63,79,84,96,98,106] (Supplementary Materials Table S1). As yet, no measurements are accessible for the bladder, genital organs (testis/epididymis, vesicular gland, scent gland, ovary, uterus), and the femur, a representative of long bones.

### 3.1. Npc1^+/+^ Control Wild-Type Mice Showed Normal Mouse Organ Weights

The organ weights of the *Npc1*^+/+^ wild-type mice lie in a range comparable with the organ weights dealt with for BALB/C mice [63,84,96,98,106] (Supplementary Materials Table S1). Although most studies described organ weights relative to body weight [28,54,63,68,80,84,97,103,107,111,112,114,116], a few reports gave absolute organs weights [63,79,84,96,98,106] (Supplementary Materials Table S1). For instance, the absolute liver weight in *Npc1*^+/+^ mice was 1.0 g [63,79,84,96,98,106]; the absolute spleen weight in *Npc1*^+/+^ mice was 0.10 g [106]; the absolute lung weight was 0.15 g [106].

Only Xie et al. [102] described the absolute liver weight for both genders separately and reported that the absolute liver weight in male *Npc1*^+/+^ mice was 1.32 ± 0.04 g and in female *Npc1*^+/+^ mice was 1.11 ± 0.03 g. The authors also determined the body weights in both genders separately: the body weight in male *Npc1*^+/+^ mice was 23.1 ± 0.3 and in female *Npc1*^+/+^ mice, 19.6 ± 0.2 g. These data correspond well with our results in female and male *Npc1*^+/+^ mice: the absolute liver weight in male *Npc1*^+/+^ mice was 1.549 ± 0.070 g and in female *Npc1*^+/+^ mice was 1.160 ± 0.038 g. The respective body weight in male *Npc1*^+/+^ mice was 26.601 ± 0.535 and in female *Npc1*^+/+^ mice was 20.558 ± 0.359 g.

Interestingly, we confirm literature data on gender-specific differences in the absolute weight of the adrenal gland. As mentioned by Hedrich [117], we also found higher weights in females compared with males (*p* < 0.001, Table 1).

### 3.2. Absolute Organ Weights of Npc1^+/+^ and Npc1^−/−^ Mice Mostly Differed in Both Genders

Compared with male *Npc1^+/+^* mice, the organ weights of the heart, liver, kidney, bladder, testis, vesicular, and scent glands of male *Npc1^−/−^* mice were lower, and femur length was shorter (Table 1). Furthermore, Xie et al. [102] found that the absolute liver weight of male *Npc1^+/+^* mice at 1.32 ± 0.04 g was lower than that of male *Npc1^−/−^* mice at 1.39 ± 0.04 g. Similarly, the absolute liver weight of the female *Npc1^+/+^* was lower than that of the *Npc1^−/−^* mice at 1.11 ± 0.03 g and 1.26 ± 0.05 g respectively. No significant differences were found in the weights of the spleen, adrenal gland, or in stomach volumes. Somewhat differing data were found in females: heart, lung, kidney, adrenal gland, ovary, and uterus of the female *Npc1^−/−^* mice had lower weights, and femur length was shorter (Table 1). No significant differences were found in the weights of the liver, spleen, or in stomach volumes (Table 1).

Seemingly, organ weights of the None mice groups can be roughly divided into two groups: (i) smaller organs were found in smaller mice, or (ii) organ weights were comparable, although mice differed considerably in body weight.

**Figure 15:**
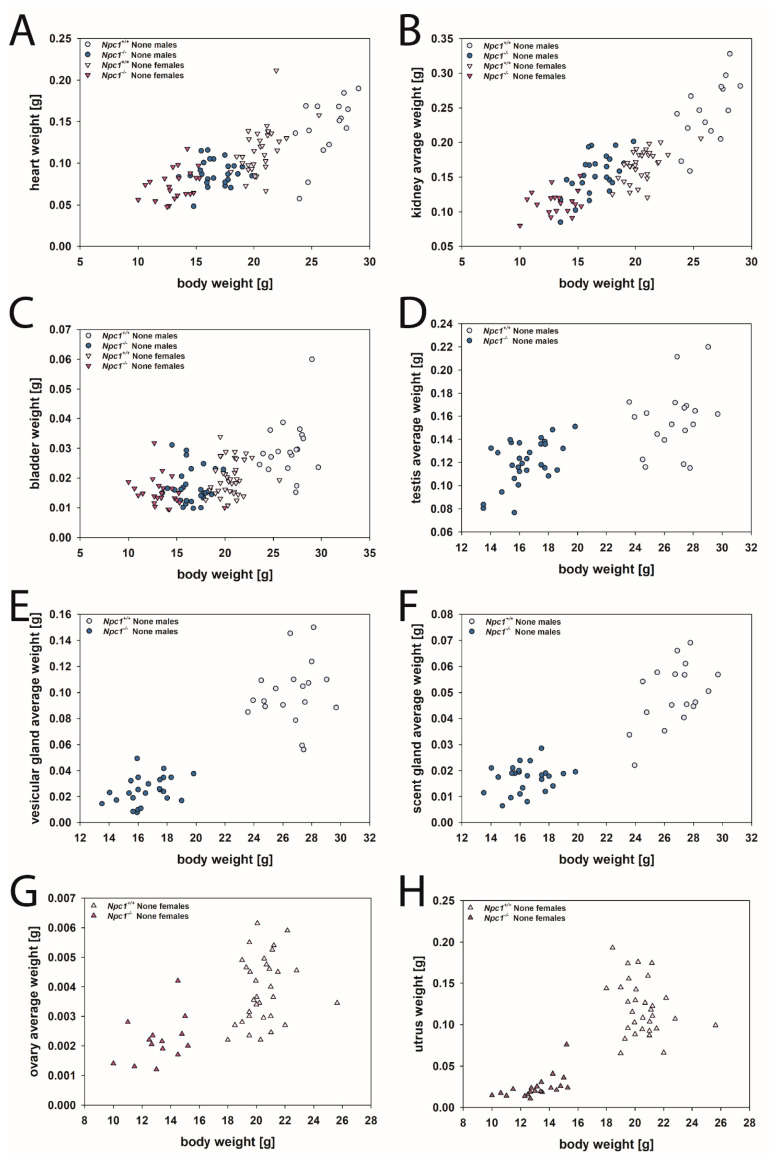
Organ weight in relation to body weight of individual *Npc1*^−/−^ and *Npc1^+/+^* mice of the None groups: (**A**) heart, (**B**) kidney, (**C**) bladder, (**D**) testis, (**E**) vesicular gland, (**F**) scent gland, (**G**) ovary, (**H**) uterus.

As shown in Figure 15, irrespective of gender and genotype, smaller organ weights were found in the heart (Figure 15A), kidney (Figure 15B), bladder (Figure 15C), testis (Figure 15D), vesicular gland (Figure 15E), scent gland (Figure 15A), ovary (Figure 15A), and uterus (Figure 15H) in mice with smaller body weights. It can be assumed that during the specific development of the heart, kidney, and bladder, changed cholesterol metabolism played a subordinate role. Interestingly, hormone-producing and hormone-dependent organs were massively underdeveloped in both genders of *Npc1^−/−^* mice (Figure 15D-H). The findings indicate that the infertility of *Npc1^−/−^* mice are reflected in the morphology. Steroid hormones are vital bioactive metabolites derived from cholesterol synthesized in the endoplasmic reticulum and mitochondria [118,119,120], which are divided according to their function and structure into glucocorticoids, mineralocorticoids, estrogens, progestins, androgens, and neurosteroids [121,122]. Because of mitochondrial abnormalities associated with NPC, an abnormal steroid hormone metabolism may be expected [59,123,124,125,126,127,128,129,130]. The *Npc1^−/−^* gene is important for the normal development of reproductive functions, illustrated by the fact that both Npc1^−/−^ affected males and females are sterile and have important histological abnormalities in the gonads [125,128]. Gévry et al. [128] showed that female BALB/c*^npcnih^^−/−^* mice are infertile with underdeveloped ovarian follicles, reduced steroidogenesis, no ovulation, and no corpora lutea. The results of this study provided strong evidence for the view that infertility in the BALB/c*^npcnih−/−^* mice is attributed to a maturation failure of the ovarian follicle to the great antrum and preovulatory stages. This leads to disruption of the cascade of ovarian and pituitary hormone secretion and prevents normal heat cycles.

Mice with a spontaneous mutation in the *Npc1* have been described as infertile [131]. The absence of the functional Npc1 causes abnormalities in spermatogenesis and deregulation of cholesterol homeostasis in the seminiferous tubules. Reducing cholesterol levels is crucial for normal sperm function [132], suggesting that balanced cholesterol levels of the sperm membranes are required for male fertility. The decrease in testosterone synthesis in *Npc1^−/−^* mice suggests that the disrupted cholesterol trafficking in NPC might also disrupt neurosteroidogenesis [59].

The work of Akpovi et al. [125] extends the findings from other studies that reported decreased testosterone production by Leydig cells in *Npc1^−/−^* mice [131]. This was seemingly not due to insufficient precursor availability of cholesterol [123] but to reduced stimulation of the pituitary gland [133], which was corrected by the expression of Npc1 in the glia [134]. Abe et al. [127] analyzed the metabolic changes of steroid hormones in the NPC model and wild-type cells and developed a simultaneous steroid hormone analysis method using LC-MS/MS, which allows for a deeper understanding of NPC pathophysiology and their involvement in mitochondrial steroid hormone production. It was found that testosterone, androsterone, progesterone, and estrone levels were significantly reduced in the NPC model cells [127].

**Figure 16:**
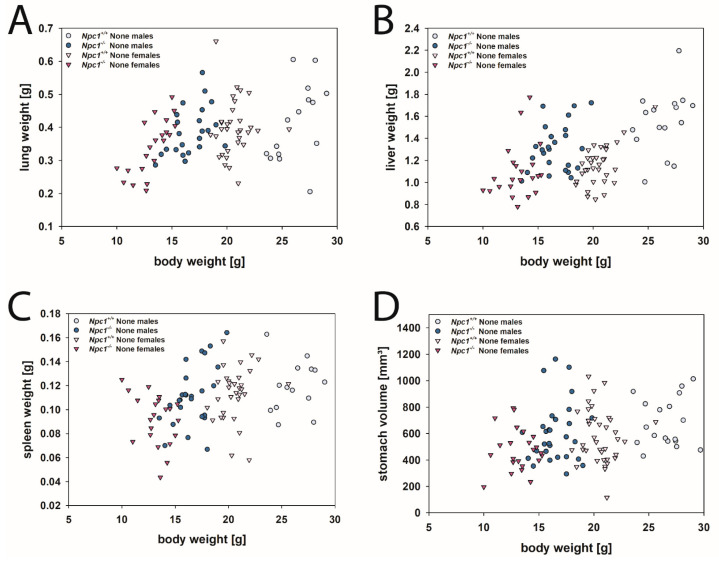
Organ weight in relation to body weight of individual *Npc1*^−/−^ and *Npc1^+/+^* mice of the None groups: (**A**) lung, (**B**) liver, (**C**) spleen, and (**D**) volume of the stomach.

The organ weights of the lung, liver, and spleen were in a comparable range, irrespective of mice gender and *Npc1* gene expression (Figure 16A–C). Our data corroborate published measures (Supplementary Materials Table S1).

Systematic studies dealing with gender-specific organ weights of *Npc1^+/+^* and *Npc1^−/−^* mice are rare. To our knowledge, only Xie et al. [102] studied both genders separately, stating that in both *Npc1^+/+^* and *Npc1^−/−^* mice, females had lower liver weights than males. The absolute liver weight was 1.32 ± 0.04 g in male *Npc1*^+/+^ mice and 1.11 ± 0.03 g in female *Npc1*^+/+^ mice. The respective body weights were 23.1 ± 0.3 g in male *Npc1*^+/+^ mice and 19.6 ± 0.2 g in female *Npc1*^+/+^ mice. These data correspond well with our results in female and male *Npc1*^+/+^ mice: the absolute liver weight in male *Npc1*^+/+^ mice was 1.549 ± 0.070 g and in female *Npc1*^+/+^ mice, 1.160 ± 0.038 g. The respective body weight in male *Npc1*^+/+^ mice was 26.601 ± 0.535 g and in female *Npc1*^+/+^ mice, 20.558 ± 0.359 g. All other available studies with weight data examined gender-mixed groups in a different number of organs.

**Figure 17:**
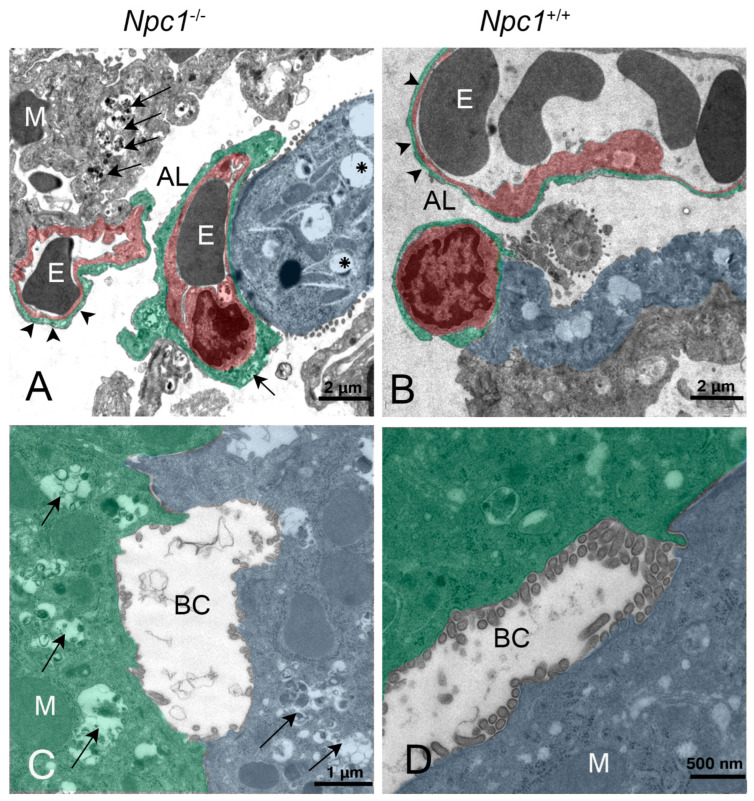
Transmission electron micrographs of the lung (**A**,**B**) and liver (**C**,**D**) of male *Npc1^−/−^* (**A**,**C**) and *Npc1^+/+^* (**B**,**D**) mice. (**A**) Myelin-like inclusions (arrows) occur in endothelial cells (colored in red), enlarged portions of type I pneumocytes (green, arrowheads) of the blood-air barrier, and macrophages (M). Type II pneumocytes (blue) often contain large secretory vesicles (*) void of lipophilic substances such as a surfactant. Al, alveolar space; E, erythrocyte. (**B**) Alveolar substructures in a wild-type mouse do not contain pathologic lipophilic deposits. (**C**) Two adjacent hepatocytes (green and blue, respectively) form a biliary capillary (**B**,**C**) with multiple lipophilic inclusions (arrows) of late Golgi stacks/lysosomes. (**D**) Similar aspect of two hepatocytes in a wild-type mouse with normal morphology. M, mitochondrion; BC, bile capillary.

It can be assumed that, especially in the lung and liver tissue of *Npc1^−/−^* mice, the sometimes massive intracellular deposits of myelin-like inclusions seen in electron micrographs (Figure 17A,C) were responsible for the relatively higher organ weights of the mutants.

**Figure 18:**
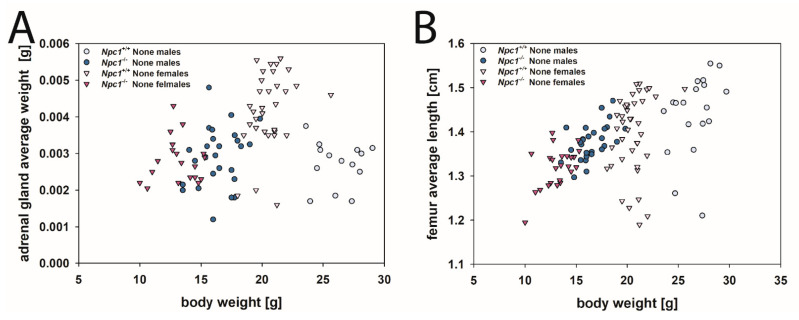
Organ weight of the adrenal glands (**A**) and femur length (**B**) in relation to body weight of individual *Npc1*^−/−^ and *Npc1^+/+^* mice of the None groups.

**Figure 19:**
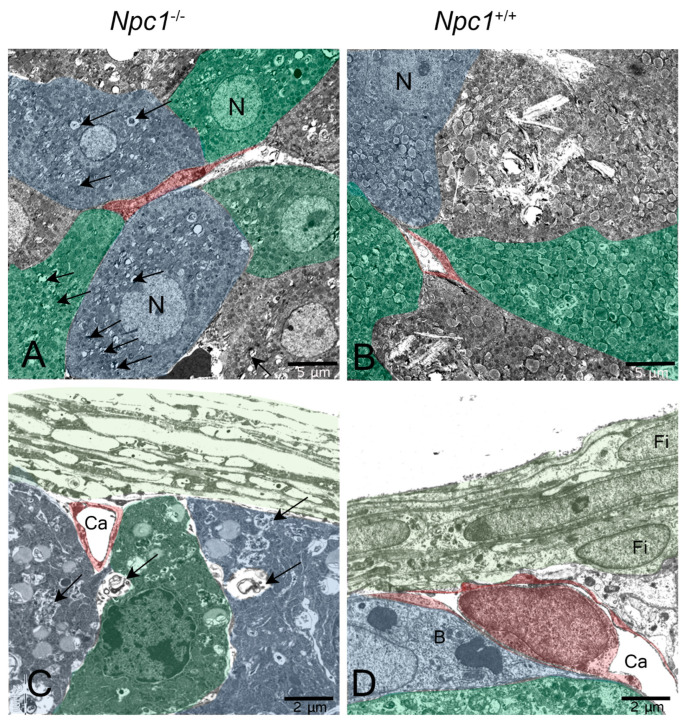
Transmission electron micrographs of the adrenal gland of male *Npc1^−/−^* (**A**,**C**) and *Npc1^+/+^* (**B**,**D**) mice. (**A**) Five adjacent adrenocortical cells are grouped around a capillary (red). Numerous myelin-like inclusions are visible (arrows). Secretory vesicles appear normal. N, nucleus. (**B**) In a wild-type animal, adrenocortical cells do not contain such pathologic deposits. (**C**) Subcapsular adrenocortical blastema cells of a mutant animal with many myelin-like deposits, some of which are shed in an enlarged intercellular space. An endothelial cell is colored red. Fibroblasts of the capsule in light green. (**D**) Normal adrenocortical blastema cells (B, green and blue) and an endothelial cell (red). Fi, capsule fibroblasts; Ca, capillary.

A conspicuously gender-different result was found for the weights of the adrenal gland. In males, the None group *Npc1*^−/−^ had weights in the range of the respective wild-types (Table 1, Figure 7A and Figure 18A). One reason could be the clear occurrence of intracellular and extracellular myelin-like deposits in *Npc1*^−/−^ mice (Figure 19A,E), not found in the respective wild-types (Figure 19B,D). However, in female *Npc1*^−/−^ mice of the None group, the organs were significantly lighter than in the respective female *Npc1*^+/+^ mice (Figure 7B and Figure 18A).

Concerning the femur lengths, it can be seen in Figure 18B that mice with smaller body weights generally had shorter femurs, irrespective of gender and genotype. Femur lengths of the four None groups were found in the identical descending order as the respective body weights: male *Npc1*^+/+^ mice, female *Npc1*^+/+^ mice, male *Npc1*^−/−^ mice, and female *Npc1*^−/−^ mice. The femur, as a representative of a long limb bone, appears to be primarily dependent on body weight for its length and not further dependent on cholesterol metabolism.

### 3.3. Relative Organ Weights of Npc1^+/+^ and Npc1^−/−^ Mice of the None Groups Partly Differed in Both Genders

The relative organ weights of the None groups in the *Npc1*^−/−^ mice, calculated as percent of the respective body weights, differed considerably between organ and gender from the wild-types. In males, the relative weights of the heart, kidney, and bladder did not differ between *Npc1^+/+^* and *Npc1^−/−^* mice; in females, besides the heart, kidney, and bladder, it was between the spleen and adrenal glands also.Other organs of *Npc1*^−/−^ mice had a higher or lower relative weight compared with *Npc1^+/+^* mice: in male *Npc1*^−/−^ mice, relatively higher weights were found in the lung, liver, spleen, adrenal gland, and testis, and only in the lung and liver in females. Relatively lower weights were found in *Npc1*^−/−^ males in vesicular and scent glands and in females in the ovary and uterus. It can be speculated that the organs which have relatively higher organ weights in *Npc1^−/−^* mice contain lipid deposits that were not found in wild-types. With the exception of the testes, all other genital organs of both genders studied (vesicular and scent glands, ovary, uterus) had disproportionately small weights. One reason could be the massive disturbances of the sexual hormone system, depending on the starting product cholesterol [59,123,124,125,126,127,128,129,130].

### 3.4. Absolute Organ Weights of Npc1^+/+^ Mice Were Mostly Left Unchanged in Both Genders by the Applied Drugs

Administration of the various drugs to *Npc1^+/+^* mice resulted in small but significant changes in organ weight compared to the None group. In male mice, MIGLU induced an increased bladder weight (+13.8%), whereas COMBI (−4.7%) and HPßCD (−5.4%) reduced femur length. Even fewer organs were affected by the drugs in *Npc1^+/+^* females: MIGLU induced a decreased adrenal gland weight (−20.8%), and following HPßCD1x the kidney (−18.0%) and the uterus were lighter (−35.7%). It can be inferred that the drug-induced changes in cholesterol metabolism had only limited effects on normal mouse development.

### 3.5. Absolute Organ Weights of Npc1^−/−^ Mice Were Partly and Differently Increased in Both Genders by the Applied Drugs

**Figure 20:**
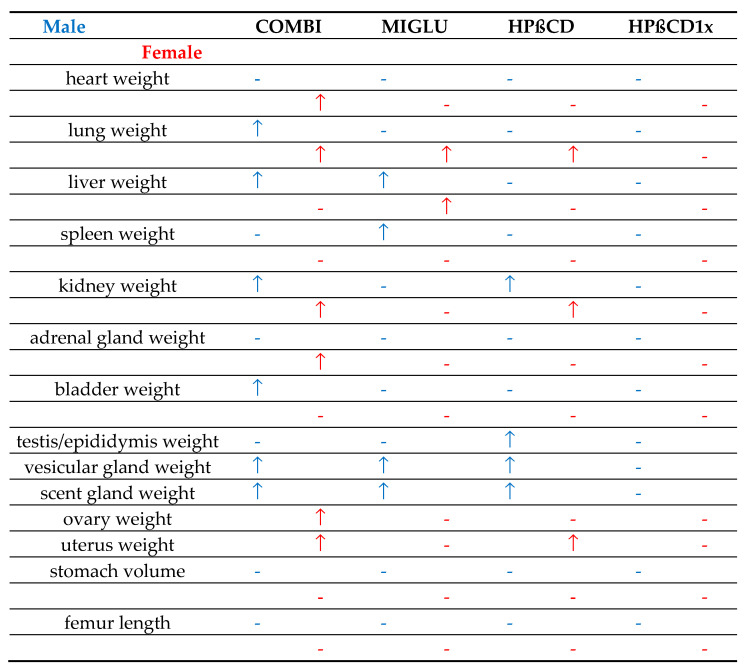
Changes of the absolute organ weights, volumes, or lengths, induced by COMBI, MIGLU, HPßCD, and HPßCD1x in male and female *Npc1^−/−^* mice. Significant amelioration (↑) or no significant change (-) compared with the respective None groups.

Regardless of which drugs were used, the body weights of male and female *Npc1*^−/−^ mice increased significantly (Figure 1) compared with the None groups. In *Npc1^−/−^* mice of both genders, the absolute organ weights were never decreased by the applied drugs (Figure 20). All drugs generally improved the health of these mice by at least in part disrupting the pathological cholesterol metabolism. The effects of the various drugs on the weights of the investigated organs, however, were quite different.

Except for a slightly increased body weight, in no absolute organ-specific measurement of male and female *Npc1^−/−^* mice, did HPßCD1x treatment have a significant effect (Figure 20). It can be speculated that a single dose of HPßCD at P7 is too little to influence these parameters. This partly contradicts the results of Liu et al. [4,64] found in gender-mixed mice groups, who state that administration of a single dose of HPßCD1x at P7 increased the lifespan of *Npc1^−/−^* mice, and at P49 still reduced cholesterol in the liver, kidney, and spleen.

**Figure 21:**
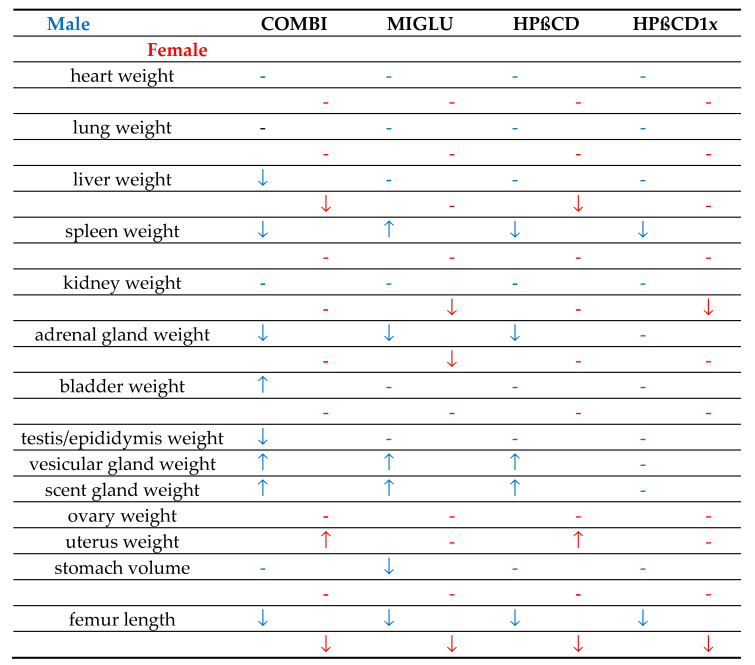
Changes of the relative organ weights, volumes, or lengths, induced by COMBI, MIGLU, HPßCD, and HPßCD1x in male and female *Npc1^−/−^* mice. Significant increase (↑), decrease (↓) or no significant change (-) compared with the respective None groups.

**Figure 22:**
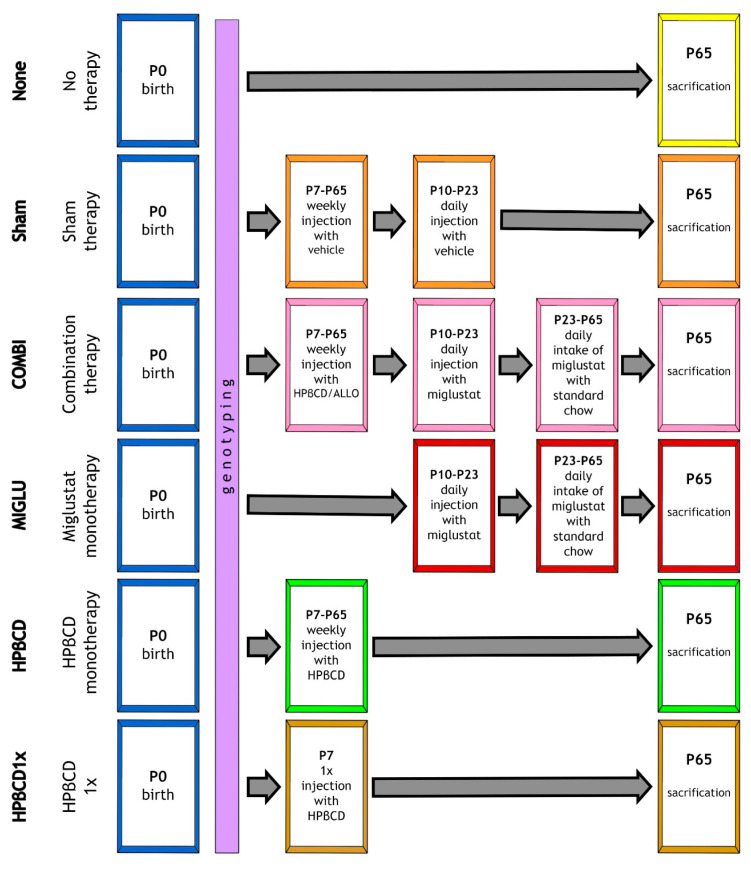
Timeline of drug administrations for all experimental groups. At P7 and thenceforth, Npc1 mice were injected weekly with allopregnanolone (25 mg/kg; Sigma Aldrich, St. Louis, MO, USA) dissolved in HPßCD (4.000 mg/kg, i.p.; Sigma Aldrich). At P10 and until P23, animals were injected daily with miglustat (300 mg/kg, i.p.; Zavesca; Actelion Pharmaceuticals, San Francisco, CA, USA). From P23 onward, animals were fed with miglustat included in standard chow (1.200 mg/kg per day) until termination. Mice of the Sham groups were injected with the respective amounts of 0.9% NaCl according to the treatment plan of the combination-treated group. Abbreviations used for the experimental groups are given in bold on the left side.

Chronic treatment with HPßCD, however, had a massive effect (Figure 20 and Figure 21) started at P7 (Figure 22). Our data agree well with the results of Davidson et al. (2009), describing a significantly improved health status in the NPC1 disease mouse model. HPßCD significantly limits cholesterol levels and ganglioside storage in neurons of young *Npc1^−/−^* mice [40]. Tanaka et al. [66] described that chronic treatment with 1000, 2000, or 4000 mg/kg HPßCD1 (subcutaneously, once a week) significantly improved the survival of *Npc1^−/−^* mice. Ramirez et al. [68] demonstrated that only weekly treatment with 4000 mg/kg) s. c. HPßCD prevents hepatosplenomegaly in *Npc1^−/−^* mice. Moreover, Lopez et al. [103] showed that systemic administration of HPβCD, starting in early neonatal life, diminishes unesterified cholesterol accumulation in most organs, slows disease progression, and extends lifespan.

With the exception of absolute stomach volume and femoral length, which showed no significant drug-related changes, all other organs increased their weight in at least one sex as part of one therapeutic scheme (Figure 22). COMBI application increased weights in six male and six female organs, MIGLU in four males and two females, and HPßCD in four male and three female ones (Figure 20). More than half of the male and female organs in which COMBI had a beneficial effect (*n* = 12) were also benefited by HPßCD treatment (*n* = 7). Thus, it can be assumed that the greater part of the COMBI effect is due to HPßCD.

### 3.6. Relative Organ Weights of Npc1^−/−^ Mice Were Differently Influenced in Both Genders

Studying the weights of organs in relation to body weight is often used in the *Npc1* literature. It gives a reliable measurement to indicate whether an organ is smaller, equal, or larger in relation to body weight [54,92,94,97,98,110,114] (Supplementary Materials Table S1). Organs that lost or gained relative weight appeared to be affected by treatment differently than the whole animal.

Taking male and female *Npc1^−/−^* mice together, in the 92 organ measurements, the relative weights in 60 cases were not significantly different from the None groups (Figure 21). For 22 measurements, the relative organ weights decreased, and for 10, they increased compared with the None groups (Figure 21).

Because we are not aware of any study that has published the effects of the drugs used in *Npc1^−/−^* mice on organ-specific weights in a gender-specific manner, the publications cited in this paragraph refer to gender-mixed mice. Comparable to Figure 21, Ebner et al. [54] showed that both COMBI and HPßCD monotherapy (injected weekly with HPβCD (4000 mg/kg starting at P7) significantly reduced the liver-to-body-weight (LW/BW) ratio in *Npc1^−/−^* mice and reached the values found in sham-treated *Npc1^+/+^* mice.

In the study of Lopez et al. [103], *Npc1^−/−^* mice and their *Npc1^+/+^* controls were given four weekly subcutaneous injections of either saline or HPβCD (4000mg/kg bw) from 49 days of age and studied at P77. When compared with their *Npc1^+/+^* controls at P49, Npc1 mutant mice exhibited the prototypical relative organomegaly of the liver, spleen, and lung. The enlargement of the liver seen in the 49-day-old *Npc1^−/−^* mice persisted in the P77 mutants given saline but was substantially diminished in their counterparts receiving HPβCD. The changes in relative spleen weight paralleled those of the liver, whereas relative kidney weights did not change with HPβCD treatment. In the case of the lungs, relative weights were consistently greater in the *Npc1^−/−^* mice but otherwise did not change as a function of age or treatment. Although Lopez et al. [103] used a late-onset therapy, their results tend to agree with ours.

Neßlauer et al. [116] analyzed the organ-to-body weight ratio in the spleen (SW/BW) after COMBI treatment. Consistent with our results, their mutant *Npc1^−/−^* mice showed increased spleen weight and increased lipid accumulation that could have been avoided by COMBI treatment.

Ramirez et al. [68] injected *Npc1^+/+^* and *Npc1^−/−^* mice with saline or a dose of HPβCD (4000 mg/kg) subcutaneously at P7 and every week thereafter until mice were studied as young adults at P49. The *Npc1^−/−^* mice that received saline alone exhibited significant enlargement of the liver, spleen, and lung but not of the other organs so far analyzed. Weekly treatment with HPβCD prevented this hepatosplenomegaly. We found comparable results in these three organs but very different results in many other organs after using different treatment strategies (Figure 20 and Figure 21).

#### 3.6.1. Relative Organ Weights of Npc1^−/−^ Mice Were Not Significantly Changed in 60 Cases

The majority of organs did not show significant drug-induced changes in their relative weights. This means that these organs changed their weight proportionally to the respective body weight, independent of the treatment used. Drug effects were comparable in these organs and throughout the body. Correspondingly, larger organs were found, for example, in the hearts and lungs of heavier Npc1^−/−^ mice. Concerning the lungs, Lopez et al. [103] showed in the NPC mouse model that, even after systemic administration of HPβCD, the relative lung weights were consistently greater in the Npc1^−/−^ mice. They did not change as a function of treatment. According to our results, the same held true for the heart in all treatment groups.

#### 3.6.2. Relative Organ Weights of Npc1^−/−^ Mice Were Significantly Decreased in 22 Cases

Differentiated results were found in the soft tissue organs liver, spleen, kidney, and adrenal gland, which had relatively reduced organ weights in some drug-treated groups either in both sexes or in only one sex. Drug treatment in these organs resulted in decreased pathological lipid deposits, so the organs were lighter than expected, considering a proportionate organ and body growth.

H&E staining of Sham-treated, COMBI-treated, or mono-treated with HPβCD (4000 mg/kg starting at P7) Npc1^+/+^ mice showed normal liver morphology and normal microvascular integration. In contrast, liver tissue from sham-treated Npc1^−/−^ mice showed necrosis. Moreover, the liver architecture was characterized by lipid accumulation in hepatocytes—analog to Figure 17C—and frequent invasion of histiocytic foam cells into sinusoids. Following COMBI therapy and monotherapy with HPβCD (4000 mg/kg starting at P7), Npc1^−/−^ mice showed an improvement in liver morphology and less necrosis but still some fat deposits [54].

Lopez et al. [103] also found large numbers of foamy, lipid-laden macrophages in the livers of Npc1^−/−^ mice who were given saline compared with their Npc1^+/+^ littermates. In contrast, in the 91-day-old mice administered with HPβCD, there was a significant reduction in the presence of these macrophages [103].

H&E staining of Sham-treated or COMBI-treated *Npc1^+/+^* spleen showed normal morphology and a regular lymphoid follicular architecture. In contrast, spleen tissue from sham-treated *Npc1^−/−^* mice showed significant morphological differences due to the infiltration of foam cells, which strikingly alter the splenic architecture by displacing the lymphoid follicles. In addition, this phenomenon was remarkably reduced in COMBI-treated *Npc1^−/−^* mice showing fewer foam cells, generally resembling the *Npc1^+/+^* phenotype [116].

Ramirez et al. [68] showed, in a multi-organ study, that in untreated P49 *Npc1^−/−^* mice, numerous lipid-laden macrophages were scattered throughout the liver. At the same time, this infiltrate is almost completely absent in HPβCD-treated animals. Even after 160 days of treatment, the architecture of the liver in *Npc1^−/−^* mice was essentially normal, apart from occasional accumulations of macrophages in a pericentral distribution. Histologically, the liver was completely normal in *Npc1^+/+^* animals treated with weekly HPβCD. However, the lungs behaved differently. In the P49, untreated *Npc1^−/−^* mice displayed small clusters of macrophages scattered throughout the alveoli of the lungs—as seen in the electron micrograph Figure 17A—and similar clusters were still found after treatment with HPβCD. This progressive infiltration continued even with weekly HPβCD treatment until, at P160, accumulations of lipid-laden macrophages filled many of the alveolar spaces. Notably, the lungs appeared completely normal in the *Npc1^+/+^* mice treated weekly with HPβCD. Finally, renal architecture was essentially normal in the untreated *Npc1^−/−^* mice, but vacuolation was noted in some tubular epithelia after administration of HPβCD, as previously reported [68].

In line with histological results showing lower drug-induced lipid storage in various organs, weight analysis revealed lower relative organ weights for the liver, spleen, and adrenal gland after MIGLU, HPβCD, and COMBI.

The significant decrease in relative femoral lengths in all four drug-treated groups of both sexes compared with the None groups is likely caused by constant femur measurements in these mice in combination with significantly increased drug-induced body weights.

#### 3.6.3. Relative Organ Weights of Npc1^−/−^ Mice Were Significantly Increased in 10 Cases

The organs belonging to the group of secondary sex organs, with the exception of the bladder in the *Npc1^−/−^* COMBI-treated males, increased their relative weight significantly. It can be speculated that a partly drug-related normalization of steroid hormone levels increased the growth and development of these hormone-dependent organs in particular. It is well known that the steroid hormone levels of the *Npc1^−/−^* None groups significantly decrease compared with the wild-types [59,123,124,125,126,127,128,129,130]. However, it must be noted that the verification of a hypothetical drug-induced increase in steroid hormone concentrations in *Npc1^−/−^* mice is still pending.

### 3.7. Drug-Specific Effects on Absolute and Relative Organ Weights of Male and Female Npc1^−/−^ Mice

Comparing the significant effects of the various treatments on organ weights of *Npc1^−/−^* mice, it can be summarized that, concerning absolute weights, the COMBI effects were seen in 12 (6 in males, 6 in females), the MIGLU effects in 6 (4 in males, 2 in females), and the HPβCD effects in 7 (4 in males, 3 in females) measurements (Figure 20). Concerning relative organ weights, significant changes were found in 11 measures (8 in males, 3 in females) after COMBI, 9 (6 in males, 3 in females) following MIGLU, and 8 (5 in males, 3 in females) following HPβCD treatment (Figure 21).

### 3.8. Organ Weights of Npc1^−/−^ Mice Depend on Various Parameters and Are Partly Influenced by Pharmacological Treatment Approaches

Specific organ weights are the result of a complex interplay of various parameters. Primarily, organ weight is dependent on body weight: heavier mice have heavier organs. Weight is also influenced by the sex of the mouse: females have smaller organs than males, possibly due to their smaller body weight and/or hormonal status. The mutant *Npc1* gene affects weight by intracellular accumulation of unesterified cholesterol, sphingomyelin, phospholipids, glycosphingolipids, and GM2 and GM3 gangliosides in late endosomes/lysosomes, endoplasmic reticulum and the Golgi apparatus. The extent of the deposits varies from organ to organ, and so do the specific organ weights. Because the metabolism of cholesterol as the precursor for the synthesis of sex hormones is massively disturbed, steroid hormone levels of *Npc1^−/−^* mice are significantly decreased compared with wild-type, resulting in hampered organ development and weight. Interventions in cholesterol metabolism by MIGLU, HPβCD, and COMBI treatments have a positive effect on clinical symptoms in *Npc1^−/−^* mice, leading to reduced lipid accumulations in organs and to a still hypothetical drug-induced increase in steroid hormone concentrations in *Npc1*^−/−^ mice. It is hypothesized that the decreased relative weights of thoracic and abdominal organs are due to drug-induced decreased lipid accumulation. The increased relative weights of sex organs are due to the drug-induced normalization of steroid hormone concentrations.

## 4. Materials and Methods

### 4.1. Animals

All animal procedures were approved by the local authorities (Landesamt für Landwirtschaft, Lebensmittelsicherheit und Fischerei des Landes Mecklenburg-Vorpommern; approval ID: 7221.3-1.1-030/12, 14 June 2012). All institutional guidelines for animal welfare and experimental conduct were followed, and all efforts were made to minimize suffering.

Heterozygous *Npc1^+^*^/−^ mice breeding pairs of Npc1 mice (BALB/cNctr-Npc1^m1N^/-J) were obtained from Jackson Laboratories (Bar Harbor, ME, USA) for generating homozygous *Npc1*^−/−^ mutants and *Npc1*^+/+^ control wild-type mice. Experimental animals were maintained under standard conditions with free access to food and water with a 12 h day/night cycle, a temperature of 22 °C, and a relative humidity of about 60%. Genotypes were determined by postnatal day P7 by PCR analysis of tail DNA as previously described [135,136]. *Npc1*^−/−^ mutants and *Npc1*^+/+^ wild-type controls of both sexes were used for different therapeutic treatment schedules. Fixed cadavers, still containing most organs, from different studies were collected from 2012 to 2019. Altogether, 176 wild-type mice (103 females, 73 males) and 175 mutant mice (92 females, 83 males) were involved in this study. The exact numbers of animals investigated in the various groups are listed in Table 2.

### 4.2. Treatment

We used 24 different animal treatment subgroups: male *Npc1*^+/+^ mice, female *Npc1*^+/+^ mice, male *Npc1*^−/^*^−^* mice, female *Npc1^−/−^* mice, each group with six subgroups: (i) no therapy (None), (ii) vehicle injection (Sham), (iii) combination of MIGLU, ALLO, and HPßCD (COMBI), (iv) MIGLU alone (MIGLU), (v) HPßCD alone starting at P7 and repeated weekly throughout life (HPßCD), and (vi) HPßCD alone given only once at P7 (HPßCD1x) (Figure 22).

Combination therapy (COMBI group): Therapy started at postnatal day 7 (P7), and weekly thereafter, mice were injected with HPßCD/ALLO (25 mg/kg ALLO dissolved in 40% HPßCD) (both from Sigma-Aldrich, Munich, Germany). Additionally, from P10 until P23, mice were injected daily with MIGLU (300 mg/kg, i.p.; Zavesca; Actelion Pharmaceuticals, San Francisco, CA, USA), dissolved in saline. Beginning at P23 and until the termination of the experiments, the mice were fed standard chow, including MIGLU, with a daily dose of 1200 mg/kg.

HPßCD monotherapy (HPßCD group): HPßCD was injected starting at postnatal day 7 (P7) and weekly thereafter, in the same dose as included in COMBI (4.000 mg/kg, i.p.; Sigma Aldrich, Munich, Germany).

HPßCD1x (HPßCD1x group): These mice received only a single injection of HPßCD at P7 (4.000 mg/kg, i.p.).

MIGLU monotherapy (MIGLU group): comparable to COMBI, mice were injected daily with MIGLU (300 mg/kg, i.p.) at P10 until P23. From P23 onward, animals were fed standard chow (V1184-000, Ssniff, Soest, Germany), including MIGLU, with a daily dose of 1200 mg/kg.

Sham (Sham group): Sham-treated mice were injected following the scheme of the COMBI mice, however, omitting the drugs in the saline.

None (None group): These mice were left uninjected.

All mice were sacrificed at P65.

### 4.3. Body Weight and Anesthesia

Before sacrification, body weights were measured, and animals were deeply anesthetized with an in-house drug mixture, a diluted solution of 0.75 g ketamine hydrochloride (contained in 7.5 mL of a 10% ketamine hydrochloride ready-to-use preparation; Ketamin^®^ 10%, Bela-Pharm, Vechta, Germany) + 0.05 g xylazine (contained in 2.5 mL of a 2% xylazine ready to use preparation; Rompun^®^, Bayer, Leverkusen, Germany), and 90 mL saline.

### 4.4. Fixation of the Animals

After sacrification, animals were perfused transcardially via the left ventricle with 0.9% sodium chloride, followed by 3.7% paraformaldehyde (PFA) solved in phosphate-buffered saline (0.1 M, pH 7.4) and thereafter postfixed with Bodian’s fixans (consisting of 900 mL of 80% isopropanol, 50 mL of 37% formaldehyde and 50 mL of glacial acetic acid) or 3.7% paraformaldehyde dissolved in phosphate-buffered saline (0.1 M, pH 7.4). Fixed cadavers were postfixed and stored in 3.7% PFA until further processing.

### 4.5. Dissection of the Animals

First, the heart was separated from the lungs and removed with forceps. Thereafter, the lungs were separated from the thorax with a thin spatula. After the dissection of the stomach and spleen, the liver was removed. The kidneys and adrenal glands were then dissected. In female mice, first, the ovaries and then the uterus were separated. In male mice, first, the scent glands were exposed, followed by the removal of the vesicular glands and the testes/epididymites. Thereafter, the bladder was dissected.

In the last step, the femora were exposed and disarticulated in the hip and knee joints.

### 4.6. Determination of the Organ Weight, Size, and Volume

The organ weights were determined using a fine scale (device GR202, accuracy 0.000(0) g A&D Europe GmbH, Darmstadt, Germany). For this purpose, the organs were removed from the PFA solution and dried briefly on filter paper. In addition, the length of the femora was measured with a caliper, and the volume of the stomach was determined from its length, width, and height.

### 4.7. Electron Microscopy

After perfusion with 0.1 M phosphate buffer containing 2.5% paraformaldehyde, six Npc1^−/−^ and Npc1^+/+^ mice were postfixed in 0.1 M cacodylate buffer containing 2.5% glutaraldehyde for at least 24 h at 4 °C. Subsequently, the adrenal glands, lung, and liver were excised and kept in the same fixative. Thereafter, the specimens were osmicated, washed, dehydrated through a graded series of ethanol, and embedded in Epon 812 (Plano GmbH, Marburg, Germany). Ultrathin sections (about 70 nm) were mounted on pioloform-coated slot copper grids and contrasted with uranyl acetate (4 min), followed by lead citrate (2 min). The specimens were examined with a Zeiss EM 902 transmission electron microscope (Zeiss, Oberkochen, Germany) at 80 kV. Photographs were taken using a CCD camera (Proscan, Lagerlechfeld, Germany) and adjusted using Photoshop CS2 software (Adobe Systems).

### 4.8. Data Analysis

The results are presented as means ± SEM. In general, an overall significance level of *p* = 0.05 was used. All data were subjected to three- or two-way ANOVA. In the case of statistically significant different mean values, the Holm-Sidak approach was used for post hoc comparisons. All statistical analyses were conducted using SigmaPlot 14 Software (Systat Software, Inc., San Jose, CA 95110, USA).

## 5. Conclusions

The combination therapy of miglustat as a substrate reduction agent (inhibitor of the glucosylceramide synthase) in combination with the sterol chelator 2-hydroxypropyl-ß-cyclodextrin (reverser of the cholesterol transport defect) seemingly had an additive effect on the normalization of the cholesterol metabolism and, subsequently, on the organ weight outcomes in *Npc1^−/−^* mice, especially in the liver, spleen, adrenal gland and genital organs. The comparison of both genders reveals that for all drug effects of 92 measures taken together, absolute weights were changed in 14 cases in males and 11 in females. Respective relative weights significantly changed in 19 of the measurements taken in males and 9 in females. Remarkably, male *Npc1^−/−^* mice were more sensitive to drug treatment.

## Figures and Tables

**Table 1:** Mean values ± SEM of the evaluated parameters of untreated (None groups) male and female *Npc1^+/+^* and *Npc1^−/−^* mice.

Parameter	Male		Female	
	*Npc1* ^+/+^	*Npc1^−/−^*	*Npc1* ^+/+^	*Npc1^−/−^*
body weight [g]	26.601	16.438 ^AAA^	20.558 ^CCC^	13.544 ^BBB,DDD^
	±0.535	±0.426	±0.359	±0.445
heart weight [g]	0.143	0.088 ^AAA^	0.115	0.075 ^BBB^
	±0.007	±0.005	±0.004	±0.004
lung weight [g]	0.414	0.388	0.398	0.335 ^B,D^
	±0.028	±0.018	±0.016	±0.019
liver weight [g]	1.549	1.312 ^AA^	1.160 ^CCC^	1.097 ^DD^
	±0.070	±0.056	±0.038	±0.046
spleen weight [g]	0.120	0.112	0.114	0.093 ^DD^
	±0.006	±0.004	±0.013	±0.015
kidney average weight [g]	0.245	0.153 ^AAA^	0.167 ^CCC^	0.115 ^BBB,DDD^
	±0.008	±0.006	±0.004	±0.005
adrenal gland average weight [g]	0.0027	0.0029	0.0042 ^CCC^	0.0029 ^BBB^
	±0.0002	±0.0001	±0.0001	±0.0002
bladder weight [g]	0.0298	0.0172 ^AAA^	0.0201	0.0151 ^CCC^
	±0.0023	±0.0019	±0.0015	±0.0019
testis average weight [g]	0.156	0.120 ^AAA^	---	---
	±0.005	±0.004		
vesicular gland average weight [g]	0.100	0.025 ^AAA^	---	---
	±0.005	±0.002		
scent gland average weight [g]	0.049	0.017 ^AAA^	---	---
	±0.003	±0.001		
ovary average weight [g]	---	---	0.0039	0.0022 ^BBB^
			±0.0001	±0.0002
uterus weight [g]	---	---	0.120	0.029 ^BBB^
			±0.007	±0.008
stomach volume [mm³]	683.15	609.81	579.59	480.53 ^D^
	±54.89	±43.24	±38.40	±47.68
femur average length [cm]	1.441	1.377 ^AA^	1.398 ^C^	1.320 ^BBB,DD^
	±0.016	±0.013	±0.011	±0.014

^A^ Significant difference between male *Npc1*^+/+^ and male *Npc1*^−/−^, ^B^ significant difference between female *Npc1*^+/+^ and female *Npc1*^−/−^, ^C^ significant difference between male *Npc1*^+/+^ and female *Npc1*^+/+^, ^D^ significant difference between male *Npc1*^−/−^ and female *Npc1*^−/−^ (^x^ *p* < 0.05, ^xx^ *p* < 0.01, ^xxx^ *p* < 0.001).

**Table 2:** Number of male and female *Npc1^+/+^* and *Npc1^−/−^* mice used in the various treatment groups.

Group	Male		Female	
	*Npc1* ^+/+^	*Npc1* ^−/−^	*Npc1* ^+/+^	*Npc1* ^−/−^
None	19	30	40	26
Sham	12	13	9	12
COMBI	17	19	20	15
MIGLU	10	8	10	12
HPßCD	11	8	9	11
HPßCD1x	4	5	15	16

## Data Availability

Not applicable.

## Supplementary Materials

The following supporting information can be downloaded at: https://www.mdpi.com/article/10.3390/ijms24010573/s1. References [137,138] are cited in the supplementary materials.

## Abbreviations

ALLO	Allopregnanolone
α-CD	α-Cyclodextrin
COMBI	Combination therapy
HPßCD	2-hydroxypropyl-ß-cyclodextrin
MIGLU	Miglustat
NPC1	Niemann-Pick disease type C1, NPC1 protein
*NPC1*	*NPC1* gene
P	Postnatal day
PFA	Paraformaldehyde
SM	Sphingomyelin
