# Supplementary material for: Organ Weights in NPC1 Mutant Mice Partly Normalized by Various Pharmacological Treatment Approaches
Source: Int J Mol Sci. 2022 Dec 29;24(1):573. doi: 10.3390/ijms24010573 (PMC9820376; doi:10.3390/ijms24010573)
Supplement: Supplementary file 1 [file ijms-24-00573-s001.zip › ijms-2080189-supplementary.pdf]

Supplementary Table S1. Overview of body and organs weights of *Npc1* mice underwent different diets and treated with various drugs.

Citation	Mice Strain, gender	Treatment	Organs weight
Beltroy et al. 2005 [1]	Wild type (*Npc1*$^{+/+}$) and homozygous mutant (*Npc1*$^{-/-}$) mice were generated from heterozygous *Npc1*$^{+/-}$ animals with a BALB/c background.	The studies delineate the biochemical, histological, and molecular abnormalities that occur in the liver of the NPC mouse maintained on low levels of dietary lipid intake.<br>All animals were fed *ad libitum* a low-cholesterol rodent diet after weaning at 19 days of age. This diet had a cholesterol content of 0.016% (wt/wt) and a total lipid content of 5% (wt/wt). Measurements were made in animals at different ages, varying from 1 to 75 days.	The growth of the whole animal was quantified, and the absolute and relative weights of the liver and brain.<br>The mean weights of the *Npc1*$^{-/-}$ animals were lower than those of the *Npc1*$^{+/+}$ mice at nearly every age, and these differences became large between 49 and 75 days of age.<br>In contrast, throughout this period of growth, the mean weight of the liver in the *Npc1*$^{-/-}$ mice equalled or exceeded that seen in the control mice. These differences were even greater when relative liver weight was calculated. Relative liver size declined during the first 2 weeks of life as the mice went into a period of rapid body growth. Beyond this age, however, relative liver size increased, and this increase was greater in the *Npc1*$^{-/-}$ animals than in the control mice. After approximately 42 days of age, the liver accounted for approximately 6% of body weight in the *Npc1*$^{+/+}$ mice but 8% in the *Npc1*$^{-/-}$ animals.
Beltroy et al. 2007 [2]	Wild type (*Npc1*$^{+/+}$) and homozygous mutant (*Npc1*$^{-/-}$) mice were generated from heterozygous *Npc1*$^{+/-}$ animals with a BALB/c background.	All animals except the heterozygous breeding stock were fed ad libitum a cereal-based, low cholesterol rodent diet (No. 7001; Harlan Teklad, Madison, WI) after weaning at 19 days of age. This diet had a cholesterol content of 0.02% (w/w) and a minimum crude fat content of 4% (w/w). The breeding stock were maintained on another formulation (Harlan Teklad No. 7002) that had cholesterol and minimum crude fat contents of 0.03% (w/w) and 6.0% (w/w), respectively. In several studies, the meal form of diet 7001 was used to prepare experimental diets containing either cholesterol (0.25, 0.50, or 1.00%, w/w) or ezetimibe (Schering-Plough Research Institute, Kenilworth, NJ) (0.00125, 0.00625, or 0.0125%, w/w). These dietary levels of ezetimibe provided doses of 2, 10, and 20 mg/day/kg bw, respectively. In some experiments, ezetimibe was administered orally to pups every day before they were weaned, starting as early as the day of birth. In these cases, the ezetimibe was suspended in medium-chain triglycerides (20 mg/ml; Mead	Body weight, relative liver weight.<br><br>On the low (0.02%) cholesterol diet, relative liver weight, hepatic total cholesterol content, whole animal cholesterol content, plasma total cholesterol concentration, and transaminase levels were all significantly higher in the *Npc1*$^{-/-}$mice compared with the control *Npc1*$^{+/+}$ animals. However, as the delivery of cholesterol to the liver in the CMr was increased by feeding the 0.20% and 1.00% cholesterol diets, there were further, significant increases in relative liver weight.<br><br>Ezetimibe was first tested at doses of 2, 10, and 20 mg/day/kg body weight in *Npc1*$^{-/-}$ mice starting when they were 19 days old. Their body weight gain was not adversely affected at any dose of the drug. However, there were significant reductions in both relative liver weight and hepatic total cholesterol content, respectively, at all three doses.

		Johnson Nutritionals, Evansville, IN) and administered using an adjustable-volume Gilson Pipetman P20 pipet (Gilson, Inc., Middleton, WI). Suckling pups treated with ezetimibe before 19 days of age were weighed and administered the ezetimibe suspension (1 ml/g bw) every 24 h. The aliquot of suspension was pipeted directly onto the back of the tongue of the pup while it was held upright, and then a swallowing reflex was induced. The pups were then immediately returned to their parents. This procedure provided an approximate dose of 20 mg/day/kg bw.<br>In this study *Npc1*$^{-/-}$ mice that had received ezetimibe from birth remained on treatment for their entire lifespan, which averaged 82 days.<br>Matching *Npc1*$^{+/+}$ animals from this same study were kept on ezetimibe treatment until 112 days of age, at which time they were used for the measurement of plasma liver enzyme activities.	
Borbon et al. 2012 [3]	Npc1nmf164 is a new, ethyl-nitrosourea-induced point mutation in the Npc1 gene.	Authors originally studied liver disease in homozygous null micewith the "natural knockout"of Npc1(Npc1nih).They studied heterozygotes versus homozygous wild-type mice atN4, at which time the genetic background is >95 %BALB/cJ.<br>Mice were maintained at the University of Arizona Animal Care Facility. They were routinely maintained on regular diet (18 %kcal fat, NIH-301). Some studies were performed after two months of a high fat diet (45 %kcal fat, Diet-07021302 produced by Research Diets, New Brunswick, NJ).	Body weight in female and male, liver weight as % of body weight.<br><br>Liver and adipose tissue (gonadal fat pads) as percent of body weight were not different between groups.

Ebner et al. 2018 [4]	Heterozygous breeding pairs of BALB/cNctr-*Npc1*m1N/J (*Npc1*$^{-/-}$) mice were obtained from the Jackson Laboratories (Bar Harbor, ME, USA) for generating homozygous *Npc1*$^{-/-}$mutants and control wild type mice (*Npc1*$^{+/+}$).	Starting at postnatal day 7 (P7) and thenceforth, mice of the combi group were injected weekly with HPßCD /ALLO (25 mg/kg ALLO dissolved in 40% HPßCD in Ringer's solution, 4000 mg/kg, intraperitoneal (i.p.), HPßCD solubilizes ALLO and serves as the vehicle for ALLO; all from Sigma-Aldrich, Munich, Germany). Additionally, 300 mg/kg miglustat (N-butyldeoxynojirimycin, Zavesca®; a generous gift of Actelion Pharmaceuticals, Allschwil, Switzerland) dissolved in 0.9% NaCl solution were daily injected from P10 to P23. From P23 onwards until termination of experiments mice were fed standard chow with embedded miglustat resulting in daily intake of 1200 mg/kg miglustat. The mice receiving only HP_CD were injected weekly with HPßCD (4000 mg/kg in Ringer's solution, i.p. Sigma-Aldrich) starting at P7. Mice of the sham group were injected like those of the combi group at the various time points with respective volumes of 0.9% NaCl or Ringer's solution, and were fed with chow without drugs.	Evaluation of liver-to-body weight-ratios.<br><br>Analysis of the liver to body weight (LW/BW) ratio showed that sham-treated *Npc1*$^{-/-}$ mice had a 1.3-fold increase of LW/BW ratio when compared to sham-treated *Npc1*$^{+/+}$ mice, while both combination therapy and monotherapy ($p < 0.05$) markedly decreased LW/BW ratio and reached values found in sham-treated *Npc1*$^{+/+}$mice.
Garver et al. 2007 [5]	A breeding pair of BALB/cJ heterozygous *Npc1* (BALB/cJ Npc1NIH) mice was obtained from The Jackson Laboratory (Bar Harbor, ME).	The present study characterizes liver disease and lipid metabolism in *Npc1* mice at 35 days of age before the development of weight loss and neurological symptoms.	Body weight, liver weight, and percent liver weight.<br><br>The mouse body weight, liver weight, and percent liver weight were obtained for normal (*Npc1*$^{+/+}$), heterozygous (*Npc1*$^{+/-}$), and homozygous affected (*Npc1*$^{-/-}$) mice at 35 days of age to determine the presence of hepatomegaly.<br><br>The results indicated no significant difference in the average body weight for *Npc1*$^{+/+}$, *Npc1*$^{+/-}$ and *Npc1*$^{-/-}$ mice, although the average body weight for *Npc1*$^{+/+}$ mice (18.3 g) and *Npc1*$^{+/-}$ mice (18.3 g) were both slightly decreased compared to the average body weight for *Npc1*$^{+/+}$ mice (19.4 g). Moreover, there was no significant difference in the average liver weight for *Npc1*$^{+/+}$, *Npc1*$^{+/-}$ and *Npc1*$^{-/-}$ mice, although the average liver weight for *Npc1*$^{+/-}$ mice (0.9 g) was slightly decreased and the average liver weight for *Npc1*$^{-/-}$ mice (1.1 g) was slightly increased compared to the average liver weight for *Npc1*$^{+/+}$mice (1.0 g). With respect to the average percent liver weight of these mice, there was a significant increase in the average percent liver weight for *Npc1*$^{-/-}$mice (6.0%) compared to the average percent liver weight for *Npc1*$^{+/+}$mice (5.3%) and *Npc1*$^{+/-}$mice (5.0%).

Hong et al. 2012 [6]	NPC mice were obtained from breeding pairs of BALB/cNctr-Npc1m1N/J mice purchased from Jackson Laboratories (Bar Harbor, MA, USA).	The purpose of this study was to find the possibility of a general therapeutic effect by applying and tracking transplanted human amniotic epithelial stem cells (hAESC) in NPC mice. hAESC were administered to NPC homozygous (*Npc1*$^{-/-}$) mice via intravenous injection from 5 weeks of age; each recipient received 5 X 10 ^{5} cells every other week.	Body weight, relative weights of the liver, spleen and kidney by body weight of *Npc1*$^{+/+}$ and *Npc1*$^{-/-}$ mouse tissue, treated with hAESC and non-treated were evaluated.<br><br>The relative weights of the liver, spleen and kidney were calculated as organ weight divided by body weight. hAESC-treated *Npc1*$^{-/-}$ mice showed smaller gains in the relative weight of the liver and kidney tissue. In contrast, the spleens of hAESC-treated *Npc1*$^{-/-}$ mice were heavier.
Jelinek et al. 2010 [7]	BALB/cJ *Npc1*^{NIH}	The *Npc1* heterozygous mouse model (*Npc1*$^{+/-}$), was used to determine whether decreased *Npc1* gene dosage was associated with weight gain when fed either a low-fat (10% kcal fat) or high-fat (45% kcal fat) diet beginning at 4 weeks of age until 20 weeks of age.	Body weight, liver weight.<br><br>*Npc1*$^{+/-}$ mice had significantly increased weight gain beginning at 13 weeks of age when fed a high-fat diet, but not when fed a low-fat diet, compared to the *Npc1*$^{+/+}$ mice fed the same diet. With respect to mice fed a high-fat diet, the *Npc1*$^{+/-}$ mice continued to have significantly increased weight gain to 30 weeks of age. At this age, the *Npc1*$^{+/-}$ mice were found to have increased liver liver weight and inguinal adipose weights compared to the *Npc1*$^{+/+}$ mice. Decreased *Npc1* gene dosage resulting in decreased Npc1 protein function, promoted weight gain in mice fed a high-fat diet consistent with a gene–diet interaction.

Jelinek et al. 2012 [8]	BALB/cJ *Npc1*^{NIH}	One week after weaning (28 days of age), the female *Npc1*$^{+/+}$and *Npc1*$^{+/-}$mice were weighed and placed on either a regular/low-fat diet (18% kcal [6.2% wt.] fat, NIH-301) or high-fat diet (45% kcal [24% wt.] fat Diet-07021302) produced by Research Diets (New Brunswick, NJ). The *Npc1*$^{+/+}$and *Npc1*$^{+/-}$mice were weighed every week from 5 to 25 weeks of age. At about 30 weeks of age, the *Npc1*$^{+/+}$and *Npc1*$^{+/-}$mice fed either the regular or the high-fat diet were killed and the liver weights and the ovarian fat pad weights were determined.	Body weight, liver weight, liver weight/body weight.<br><br>No significant differences in weight curves were found between female *Npc1*$^{+/-}$ and *Npc1*$^{+/+}$mice when fed a regular diet (18% of calories from fat) although there was a trend for them to be lighter at 19–23 weeks of age. However, when placed on the high fat diet (45% of calories from fat), female *Npc1*$^{+/-}$were heavier than female *Npc1*$^{+/+}$mice from week 9 onwards and the difference had become significant from 18 weeks onwards. At 30 weeks of age, the *Npc1*$^{+/-}$mice on the high fat diet had a significantly increased (23.3%, p = 0.0022) body weight compared to *Npc1*$^{+/+}$mice fed the same high-fat diet. This was associated with increased white adipose tissue weight but not liver weight. The females on the regular diet did not show differences in these parameters.
Jelinek et al. 2013 [9]	Male BALB/cJ Npc1 heterozygous mouse (BALB/cJ Npc1$^{+/-}$) and a female C57BL/6J Npc1 normal or wild-type mouse (C57BL/6J Npc1$^{+/+)}$ were obtained from The Jackson Laboratory (Bar Harbor, ME).	The present study was performed using both BALB/cJ and C57BL/6J Npc1$^{+/+}$ and Npc1$^{+/-}$ mice to determine if decreased Npc1 gene dosage predisposes to metabolic features associated with type 2 diabetes.	Body weight, liver weight.<br><br>Since the C57BL/6J Npc1$^{+/-}$ mice have impaired glucose tolerance in the absence of a change in body weight, further characterization of C57BL/6J Npc1$^{+/+}$ and Npc1$^{+/-}$ mice was performed to define the possible mechanism. The additional results indicated no significant difference in the body weights, liver weights, or adipose weights between Npc1$^{+/+}$and Npc1$^{+/-}$ mice at 84 days of age after sacrifice. Moreover, as would be expected for no change in body weight, there was no significant difference in liver weights or adipose weights when normalized to respective body weights (data not shown). The liver lipid analysis of C57BL/6J Npc1$^{+/+}$ and Npc1$^{+/-}$ mice indicated no significant difference in the concentration of liver total cholesterol (cholesterol and cholesteryl ester) or triacylglycerol, although there was a significant increase (35%) in the concentration of liver free fatty acids among Npc1$^{+/-}$ mice compared to Npc1$^{+/+}$ mice.

Jiang et al. 2020 [10]	A colony of 36 Npc1$^{-/-}$ mice, also called the *Npc1*m1N mouse (16 females, 20 males), was developed at the Jackson Lab (JAX, Bar Harbor, ME) for this study (JAX stock #003092).	The first group treated by vehicle (Hepes bufer, pH=7.0), and the second group treated with TfRMAb-targeted THLs encapsulating the pPDGFB-*NPC1* plasmid DNA (designated TLC-200). Each mouse was treated by weekly tail vein injections of a volume of 150 μL. Te injection dose (ID) in the THL treated mice was 6 μg plasmid DNA per mouse, and the ID of the TfRMAb was 15 μg per mouse.	Body weight, brain, liver, and spleen weights in females and males mice.<br><br>Mice were monitored weekly with body weights and clinical signs (tremor, ruffed fur, yellow coat, abnormal gait).<br><br>At euthanasia, brain, liver, and spleen were removed from all mice for organ weights.<br><br>There was no difference in body weight (BW) between the treatment groups at the end of the study. The female mice had a BW of 17±2 g and 18±2 g at the start of the study in the vehicle and THL groups, respectively. The male mice had a BW of 23±2 g and 23±1 g at the start of the study in the vehicle group and THL groups, respectively. There was no statistical change in brain weight in the vehicle or THL treatment groups for either sex. However, there was a significant 42% and 37% increase in spleen weight in the THL group as compared to the vehicle group in the females and males, respectively. There was a significant 10% increase in liver weight in the male mice treated with THLs. When the spleen and liver weights were normalized by BW for each mouse, the increase in spleen weight was 37% and 33% in the female and male groups, respectively ($P<0.001$).
Li et al. 2005 [11]	Heterozygous *Npc1* mice with a BALB/c background were mated.	This study defines the functional, biochemical, and molecular events that ensue as nerve cell death occurs.<br>In most studies, the animals were weaned at 3 weeks of age onto a basal rodent diet (No. 7001 Harlan Teklad, Madison WI) that contained 0.02% (wt/wt) cholesterol. In some studies, groups of mice were placed on the same diet that had been enriched with 1.0% cholesterol (wt/wt) from the time of weaning until the end of the experiments. To first evaluate the interaction of gender and cholesterol intake, 4 groups of *Npc1*$^{-/-}$ and *Npc1*$^{+/+}$ mice were weaned onto diets containing either 0.02% or 1.0% cholesterol and observed for the duration of their lives.	Body weight, relative brain, liver, spleen, lung weights.<br><br>As is apparent, the size of the liver, spleen, and lung weights, expressed as a percent of body weight, was significantly greater in the *Npc1*$^{-/-}$ mice compared with the control animals at 7 weeks of age. In contrast, relative brain weight was similar in the *Npc1*$^{-/-}$ and *Npc1*$^{+/+}$ mice.
Li et al. 2008 [12]	The *Npc1* mice obtained from the Pentchev laboratory were on a pure BALB/c background and, subsequently, were bred	These studies investigated the role of gangliosides in governing the steady-state concentration and turnover of unesterified cholesterol in normal tissues and in those of mice carrying the *Npc1* mutation.	Organ weights: adrenal, brain, lung, kidney, stomach, spleen, small bowel, colon, liver, testis, heart, muscle; cholesterol concentrations, and cholesterol synthesis rates in control mice and in animals lacking GM2/GD2 synthase, GM3 synthase, or both GM2/GD2 and GM3 synthase.

	into a mixed 129/Sv and C57BL/6 background, which was similar to that present in the GM2/GD2 and GM3 knockout animals provided by Dr. Proia.	These various stocks of animals were used, in turn, to generate groups of littermates that were normal controls (designated Galgt$^{+/+}$/Siat9$^{+/+}$), lacked GM2/GD2 synthase activity (Galgt1$^{-/-}$/Siat9$^{+/+}$), lacked GM3 synthase activity (Galgt1$^{+/+}$/Siat9$^{-/-}$), or lacked both of these synthases (Galgt1$^{-/-}$/Siat9$^{-/-}$).	The organ weights in the animals lacking GM2/GD2 synthase were essentially the same as in the control mice, except for minor differences in the adrenal, kidney, and testis. Notably, both the absolute and relative weights of the liver (5.58 6 0.14 vs. 5.56 6 0.08% of body weight) and brain (1.94 6 0.07 vs. 1.87 6 0.05%) were not significantly different in the Galgt1$^{-/-}$ /Siat9$^{+/+}$ mice, compared with the control animals.
Lin et al. 2017 [13]	OPN (osteopontin) gene knockout (OPN-/-) mice and wild-type mice.	The present study investigated the role of OPN in cholesterol gallstone formation, focusing on its effect on intestinal absorption of cholesterol. OPN gene knockout (OPN-/-) mice and wild-type mice were respectively fed with a chow or lithogenic diet (LD) for 8 weeks. WT mice were purchased from Fudan University (Shanghai, China). OPN-/- mice in congenic background were purchased from Jackson Laboratory (Bar Harbor, ME, USA). Mice were housed at 22±2℃ and 60±10% relative humidity in a specific pathogen-free environment, with a 12:12 h light: dark cycle. WT and OPN-/- male mice between 8 and 10 weeks of age were fed a chow diet (CD) or LD (CD supplemented 15% fat, 2% cholesterol, and 0.5% cholic acid) for up to 8 weeks.	Body weight, gallbladder volume.<br><br>The body weight and gallbladder volume between two strains showed no difference.
Liu et al. 2008 [14]	Mice were lacking functional Npc1 protein (*Npc1*$^{-/-}$) on a BALB/c background. A second group of similar *Npc1*$^{-/-}$ mice was derived from heterozygous founders from the Jackson Laboratories (BALB/cNctr-*Npc1*^{m1N}/J; stock number 003092).	All mice were fed ad libitum a low cholesterol (0.02%, w/w) rodent diet or the ground meal form of this same diet containing either cholesterol (1%, w/w) or an LXR agonist (T0901317). Intake of this latter diet provided each animal with an approximate daily dose of T0901317 equal to 50 mg/kg body weight. Other groups of mice were administered a single sc. injection at 7 days of age of a 20% (in saline) solution of HPßCD (4,000 mg/kg body weight). In addition, in some experiments, ALLO (5a-pregnan-3a-ol-20-one) was added to the	Whole body animal weights and absolute and relative liver weights.<br><br>However, although both groups of mice had similar body weights, enlarged livers, and cholesterol concentrations in the liver, spleen, and lung, the plasma transaminase levels were only increased half as much in the Jackson, compared with the UTSW, animals.

		HPßCD solutions at a concentration of 1.5 mg/ml (to provide 25 mg/kg body weight).	
Lopez et al. 2014 [15]	Control (*Npc1*$^{+/+}$) and homozygous (*NCP1*$^{-/-}$) mice were generated from heterozygous (*Npc1*$^{+/-}$) breeding stock on a pure BALB/c background.	*Npc1*$^{-/-}$ and *Npc1*$^{+/+}$ mice that had not received any prior treatments were administered weekly a subcutaneous injection at the scruff of the neck of either normal saline or a 20% (w/v) solution (in saline) of 2HPβCD (Sigma-Aldrich Corp; product H107) starting when they were 49-days old (approximate dose of 4000 mg/kg bw). Matching groups of 49-day old *Npc1*$^{-/-}$ and *Npc1*$^{+/+}$ mice received their respective treatments (saline vs 2HPβCD) at 49, 56, 63, and 70 days of age. At 77 days of age these mice were anesthetized, exsanguinated, and multiple organs were taken.	Body weight and relative organ weight of the liver, spleen, kidney, lung and brain.<br><br>When compared to their *Npc1*$^{+/+}$ controls at 49 days of age, *Npc1*$^{-/-}$ deficient mice exhibited the prototypical organomegaly of the liver, spleen, and lung. The enlargement of the liver seen in the 49 day-old *Npc1*$^{-/-}$ mice persisted in the 77 day-old mutants given saline but was substantially diminished in their counterparts receiving 2HPβCD. The changes in relative spleen weight paralleled those of the liver, whereas relative kidney weights did not change with 2HPβCD treatment. In the case of the lung, relative weights were consistently greater in the *Npc1*$^{-/-}$ mice but otherwise did not change as a function of age or treatment.
Lopez et al. 2018 [16]	Heterozygous Npc1nih/nih mice (BALB/c background) were generated from breeding stock originally supplied to us by Dr. Peter Pentchev at the Developmental and Metabolic Neurology Branch, National Institute of Neurological Disorders and Stroke (Bethesda, MD)	All progenies were genotyped on or before 21 days of age, which is when they were weaned. They were fed ad libitum a cereal-based rodent chow diet (no. 7001, Envigo, Teklad, Madison, WI). This formulation had an inherent cholesterol content of 0.02% (wt/wt) and a crude fat content of 4.4% (wt/wt). It provided 13% of calories from fat and was fed as pellets in all experiments except one in which the meal form of 7001 was made to contain cholesterol at a final level of 1.0% (wt/wt). This regimen was fed to mice of the 4 specified genotypes for 15 days, starting when they were 35 days old. A comparable cholesterol-enriched diet was used in some of our previous projects with the *Npc1* mouse model. Other investigators have used a cholesterol level of 1% (wt/wt) with added fat or 2% (wt/wt) cholesterol with no other additions. For our earlier investigations of sterol metabolism in Soat2-knockout mice we used chow, either alone or containing 0.5% (wt/wt) cholesterol. All mice were housed as previously described and were studied in the fed state at an average age of ~7 wk. Historically, most metabolic studies in mice deficient in *Npc1* are	Absolute organ weight: liver, small intestine, spleen, lung, brain.<br><br>*Npc1* mutant mice fed a chow diet typically exhibit an increased mass of the liver in particular, and to a lesser extent of the spleen and lungs but not of the small intestine. These findings were replicated in the *Npc1*$^{-/-}$: Soat2$^{+/+}$ mice in the present studies, although only for the liver was the difference statistically significant. In the *Npc1* mutant mice, with or without SOAT2 function, the brain weight data were reflective of the hallmark demyelination that starts occurring before 7 wk of age in this model. The main finding is that there was a clear trend toward reduced hepatomegaly in the *Npc1*$^{-/-}$:Soat2$^{+/+}$ mice.

		done when they are no more than ~7 wk of age because their physical condition declines significantly after that age. For the parameters that the present studies focused on, particularly hepatic cholesterol levels, we and other investigators have not previously found consistent sex-related differences in either the Npc1- or Soat2-knockout models. Therefore, in the present studies, data from males and females within each genotype were pooled as noted in the figure legends.	
Lopez et al. 2020 [17]	All studies were carried out in *Npc1*$^{-/-}$ and *Npc1*$^{+/+}$ mice on a pure BALB/c background (Npc1nih) derived from heterozygous breeding stock kindly supplied by Dr. Peter Pentchev.	All animals were weaned onto a pelleted, cereal-based rodent chow diet (No. 7001, Envigo Teklad Madison, WI). This diet had an inherent cholesterol content of about 0.02% w/w and a crude fat content of about 4.4% w/w. In the case of the ezetimibe study which was done in only female mice, the compound was added directly to a powdered form of this diet at varying levels so as to provide doses of approximately 2, 10 or 20 mg/day/kg bw, starting from the day of weaning until study at 56 days of age. The cholesterol feeding study was done in male mice given powdered chow with cholesterol added to a level of 0.20% w/w. This diet was fed for 14 days starting when the mice were 35 days old. For the ontogenesis study, we tracked the changes in body and small intestine weight and intestinal cholesterol levels in male and female *Npc1*$^{-/-}$and *Npc1*$^{+/+}$ mice from 23 to 77 days, with all mice being fed the pelleted chow diet. This was also the case in subsequent experiments involving the measurement of intestinal cholesterol and triacylglycerol concentrations, or of fractional cholesterol absorption, fecal neutral sterol excretion, and the concentration of cholesterol in gallbladder bile in young adult mice. These data were obtained from a mixture of males and females in each genotypic group. The mice used for bile harvesting were fasted	Body weight, small intestine weight.<br><br>Small intestine weight in *Npc1*-deficient mice was unchanged with disease progression. While a discernible genotypic difference in body weight was evident by 56 days after birth, the same trend was not seen with small intestine weight.<br>In groups of *Npc1*$^{+/+}$ and *Npc1*$^{-/-}$ mice given a diet with a tenfold increase in cholesterol content for 14 days starting at 35 days of age, there was no discernable impact on small intestine weight in mice of either genotype.<br>Treatment with ezetimibe, a cholesterol absorption inhibitor, did not significantly change small intestine weight, but it moderately lowered the level of intestinal cholesterol sequestration at the higher doses of inhibitor.

		for 4–6 h beforehand to increase the yield of bile. In the studies involving acute treatment with 2HPβCD, male and female mice that had been maintained on the chow diet were studied in the age range of 49–51 days. They received a single bolus injection at the scruff of the neck of 2HPβCD in saline at a dose of 4000 mg/kg bw, or of saline only, and were studied 24 h later.	
Maass et al. 2015 [18]	BALB/c-*Npc1*^{nih}	At P7 and thenceforth, *Npc1*$^{-/-}$ mice were injected weekly with ALLO (25 mg/kg) dissolved in HPßCD (4,000 mg/kg, i.p.). At P10 and until P23, animals were injected daily with MIGLU (300 mg/kg, i.p.). From P23 onward, animals were fed with MIGLU as powdered chow (1,200 mg/kg per day) until termination.	Whole brain weight.<br><br>In *Npc1*$^{-/-}$ sham mice, brain weight was 0.47460.006 g. Brain weights in both of the other groups were significantly decreased (*Npc1*$^{-/-}$ sham 0.38960.005 g and *Npc1*$^{-/-}$ SRT/BPT 0.38760.004 g) compared with *Npc1*$^{+/+}$ sham (P<0.05).
Mundy et al. 2014 [19]	Cav-1-deficient mice (Cav-1$^{-/-}$), and subsequently in Cav-1$^{-/-}$ mice that also lacked the lysosomal cholesterol transporter Niemann-Pick C1 (NPC1) was also absent (Cav-1$^{-/-}$: *Npc1*$^{-/-}$).	All litters were weaned at 21 days onto a cereal-based rodent chow diet (Teklad 7001 Madison, WI). This formulation had an inherent cholesterol and crude fat content of 0.02 and 4% (wt/wt), respectively. In one study involving cholesterol-fed mice, the level of cholesterol in the diet was raised to 0.5% (wt/wt).	Body weight, relative lung weight.<br><br>In keeping with the known phenotype of Cav-1$^{-/-}$ mice, a marginally lower body weight was evident (data not shown) and this was a factor in making their relative lung weights higher than those in the Cav-1$^{+/+}$ controls. Even at 24 days of age, the Cav-1$^{-/-}$ mice had a significantly greater lung mass. The magnitude of the genotypic difference in lung weight at both 50 and 100 days was the same as that evident at 24 days. The genotype-related differences in relative lung weight irrored those seen in the absolute weight. For both the Cav-1$^{-/-}$ and Cav-1$^{+/+}$ mice at 24 days, relative lung weights were clearly greater than they were in the older mice.<br>The average body weight of the mice deficient in both Cav-1 and Npc1 at 50 days of age was the same as that of their littermates lacking only Npc1. There was a trend for the body weight of these two groups to be less than that of the mice deficient in only Cav-1, which in turn had a lower mean body weight than the Cav-1$^{+/+}$: *Npc1*$^{+/+}$ controls. There were pronounced genotypic differences in absolute lung weights, particularly for the Cav-1$^{-/-}$: *Npc1*$^{-/-}$mice. In these mice, the increase in lung mass exceeded the combined increases associated with deficiency of either Cav-1 or Npc1 alone.
Neßlauer et al. 2019 [20]	BALB/cNctr-*Npc1*m1N/J (*Npc1*$^{-/-}$) mice	The combination treatment starting at P7, includes weekly injection of mice with 2-hydroxypropyl-ß-cyclodextrin (HPßCD, 4000 mg/kg, i. p., Sigma	Spleen-to-body-weight ratios (SW/BW) of sham-treated *Npc1*$^{+/+}$, sham-treated *Npc1*$^{-/-}$, treated *Npc1*$^{+/+}$, and treated *Npc1*$^{-/-}$ mice.

		Aldrich, St. Louis, MO, United States) and allopregnanolone (Pregnan-3alpha-ol-20-one; Sigma Aldrich, St. Louis, MO, United States) (25 mg/kg allopregnanolone dissolved in 40% HPßCD in Ringer's solution). Additionally, from P10 to P22 mice were injected daily with miglustat (N-butyldeoxynojirimycin, Zavesca®; Actelion Pharmaceuticals, Allschwil, Switzerland), dissolved in 0.9% NaCl solution, 300 mg/kg i. p.). Thereafter, miglustat powder was mixed with standard chow and administered until P65, resulting in a daily intake of 1200 mg/kg miglustat. "Sham-treated" *Npc1*$^{+/+}$ and *Npc1*$^{-/-}$ mice were injected with Ringer's solution or normal saline solution following the same treatment. Animals were sacrificed at P65.	The organ to body weight ratio of spleens (SW/BW) were analysed. The evaluation of SW/BW ratio showed that sham-treated *Npc1*$^{-/-}$ (0.08868 ± 0.02956) mice had an increased SW/BW ratio compared to sham-treated *Npc1*$^{+/+}$ (0.05866 ± 0.01769) mice (p =0.250). Both treated *Npc1*$^{+/+}$ (0.11650 ± 0.03113, p = 0.036) and *Npc1*$^{-/-}$ (0.10660 ±0.03078) mice (p = 0.006) had a significantly increased SW/BW ratio compared to sham-treated *Npc1*$^{+/+}$ mice.
Parra et al. 2011 [21]	C57BL/6JNpc1/ mice and BALB/c-Npc1/ mice	Mice had free access to water and a chow diet.<br><br>To study the influence of genetic background on the expression of NPC disease in mice, authors transferred the Npc1 mutation from the BALB/c to C57BL/6J inbred background.	Brain, liver and spleen relative weights of BALB/c and C57BL/6J wild-type and *Npc1* mutant mice were measured.<br><br>To analyze to what extent the *Npc1*$^{-/-}$ mouse model mimics human disease pathology, we measured the size of the livers, spleens, and brains of 4-week-old mice. The relative liver size was significantly increased in Npc1/ mice from both genetic backgrounds in comparison to matched age controls. Interestingly, C57BL/6J- *Npc1*$^{-/-}$ mice presented a decrease in spleen size in comparison to wild type controls, which suggests dysfunction of this organ. One interesting and unexpected finding was that C57BL/6J- *Npc1*$^{-/-}$ mice had a relatively larger brain sizes in comparison to C57BL/6J- *Npc1*$^{+/+}$ mice.
Ramirez et al. 2010 [22]	Control (*Npc1*$^{+/+}$) and homozygous mutant (*Npc1*$^{-/-}$) mice were generated from heterozygous (*Npc1*$^{+/-}$) animals on a pure BALB/c background, and the pups were genotyped at 19 days of age.	Animals were fed ad libitum a cereal-based, low-cholesterol (0.02% cholesterol, 4% total fat, w/w) diet (no. 7001; Harland Teklad, Madison, WI) upon weaning. Groups of mice were administered a subcutaneous injection of a 20% (w/v, in saline) solution of 2-hydroxypropyl-β-cyclodextrin (Sigma; product H107), (4000 mg/kg body weight), during the late dark phase (09:00 hours) at the scruff of the neck. Matching mice were also injected with saline alone to serve as controls.	The relative organ weights were measured in six major tissues: brain, liver, spleen, lung, kidney, small bowel.<br><br>The *Npc1*$^{-/-}$ mice receiving only saline had significant enlargement of the liver, spleen, and lung, but not of the other organs. Weekly treatment with HPßCD prevented this hepatosplenomegaly.
Ramirez et al. 2014 [23]	Control (*Npc1*$^{+/+}$ and Npc2$^{+/+}$ and mutant	The present studies investigated how Npc1 deficiency impacts the absolute weight, lipid	Body weight, absolute lung weight, absolute whole weight of lung lobe, relative lung weight.

	($Npc1^{-/-}$ and $NPC2^{-/-}$) mice were generated from respective heterozygous breeding stock on a pure BALB/c background. The *Npc1* mice (Npc1nih) originated from a colony at the National Institutes of Health (Dr. Peter Pentchev).	composition and histology of the lungs of $Npc1^{-/-}$ mice (Npc1nih) at different stages of the disease, and also quantitated changes in the rates of cholesterol and fatty acid synthesis in the lung over this same time span (8 to 70 days of age). Similar measurements were made in $NPC2^{-/-}$ mice at 70 days. Unless studied beforehand, all mice were weaned at 19 to 21 days, and thereafter were fed ad libitum a cereal-based, low-cholesterol (0.02% cholesterol, 4% total fat, w/w) diet (No. 7001; Harland Teklad, Madison, WI).	These changes are accompanied by progressive neurodegeneration which leads to a shortened life span of around 80 to 90 days. The features of $NPC2^{-/-}$ mice parallel those lacking Npc1 but their lifespan is generally longer, depending partly on the strain of mouse. $Npc1^{-/-}$ mice show a significant increase in lung mass starting before weaning At 8 days after birth the weight of the lungs in the $Npc1^{-/-}$ mice (0.080 ± 0.003 g, n=10) was about the same as that in matching $Npc1^{+/+}$ controls (0.079 ± 0.003 g, n=12). However, at all ages after 8 days the lung mass in the $Npc1^{-/-}$ mice was consistently greater than in their $Npc1^{+/+}$ controls. It can be calculated that across the age span from 19–22 to 77–80 days, the average weight of the lungs in the $Npc1^{-/-}$ mice (0.165 ± 0.010 g) was significantly higher than in the matching $Npc1^{+/+}$ controls (0.134 ± 0.006 g, $p<0.05$). At least in mice in the age range of 57–60 days, the greater lung mass in the mutants was not confined to any one lobe although the bulk of the additional tissue was in the left and diaphragmatic lobes.<br><br>Lung weights relative to body weights in $Npc1^{-/-}$ and $Npc1^{+/+}$ from 8 to 70 days which was generally the age range over which various parameters of lung cholesterol and lipid metabolism were measured. It should be noted that after about 49 days of age the genotypic difference in relative lung weight becomes exaggerated because of the steady decrease in the body weight of the $Npc1^{-/-}$ mice in late stage disease.

Rodriguez-Gil et al. 2020 [24]	All mice used in this study were maintained on a standard Purina Prolab RMH 1800 diet. The Npc1em1Pav line (abbreviated Npc1em) was previously generated by CRISPR/Cas9 targeting of exon 21 of Npc1 on a C57BL/6J background, which caused a nine-base pair, in-frame deletion that is predicted to result in deletion of 3 amino acids, Ser1062, Asn1063, and Ile1064 (Rodriguez-Gil et al., under revision).	The authors performed the first developmental analysis of a Npc1 mouse model, Npc1em1Pav, and discovered significant fetal growth restriction in homozygous mutants beginning at E16.5.	Body weight, lung weight/body weight, heart weight/body weight, liver weight/body weight.<br><br>Normalized weights (in grams) of lung, heart, and liver in Npc1em/em mutants at birth are not significantly different from *Npc1*$^{+/+}$ and Npc1em/+ littermates. Wet weights of organs were analysed at birth and normalized to total body weight.
Santiago-Mujica et al. 2019 [25]	BALB/cNctr-*Npc1*<m1N>/J	Animals of 4-10 weeks of age were analysed for general health, motor deficits as well as hepatic and neuronal alterations with a special focus on cerebellar pathology.	Body weight, brain and liver weights.<br><br>At 6 weeks of age, the liver was weighed and the total levels of cholesterol and hepatic enzymes were measured in the liver and plasma, respectively. *Npc1*$^{-/-}$ mice showed a 1.2 fold increase in liver weight compared to WT littermates.<br><br>In order to study possible brain alterations in *Npc1*$^{-/-}$ mice, the brain was weighed and the levels of total cholesterol and Ab were measured in 6 week old mice compared to age-matched WT littermates. The weight of the cerebellum and cortex was significantly lower in *Npc1*$^{-/-}$ mice compared to WT littermates.
Schlegel et al. 2016 [26]	Male BALB/c-npc1nih*Npc1*$^{-/-}$ wild type mice (*Npc1*$^{+/+}$)	Starting at postnatal day 7 (P7) and thenceforth, mice of the COMBI-group were injected weekly with HPßCD/ALLO (25 mg/kg ALLO dissolved in 40% HPßCD in Ringer's solution, 4000 mg/kg, i.p.). Additionally, these mice were daily injected with MIGLU, 300 mg/kg i.p. from P10 to P23. From P23	Body weight, brain weight.<br><br>Mean body weight of sham-treated mice was 24.6 g (SD: 1.5 g), for COMBI-treated mice 22.5 g (SD: 1.6 g) and 24.4 g (SD: 1.3 g) for MIGLU-treated mice. Mean body weight of the COMBI-group was significantly reduced compared to the sham-group and the

		onwards mice were fed standard chow with embedded MIGLU resulting in daily intake of 1200 mg/kg MIGLU. The MIGLU-group was treated like the COMBI-group, but without HPßCD/ALLO, instead mice got vehicle. Mice of the sham-group were injected like those of the COMBI-group with the respective volumes of 0.9% NaCl or without volume and were fed with chaw without drugs.	MIGLU-group, whereas body weights of sham- and MIGLU-groups did not significantly vary ($p = 0.610$).<br><br>The brains of COMBI - (mean: 0.408 g, SD: 0.0234 g) and MIGLU-treated mice (mean: 0.426 g, SD: 0.0156 g) were significantly lighter ($p < 0.001$) compared to sham-treated mice (mean 0.449 g, SD: 0.0278 g).
Walenbergh et al. 2015 [27]	Female Ldlr$^{-/-}$ mice on a C57/Bl6 background mice	Eleven to twelve-week old female Ldlr$^{-/-}$ mice on a C57/Bl6 background were either fed regular chow (n = 10) or an HFC diet (n = 12 per HFC group with and without HP-B-CD treatment) for 12 weeks. The effects of HP-B-CD were investigated by giving weekly subcuteanous injections at the start of the HFC diet with 4000 mg per kg of body weight of 20% w/v HP-B-CD (H107, Sigma-Aldrich GmbH, St. Louis, MO, USA) (n = 12). PBS was used for control injections. The HFC diet contained 21% milk butter, 0.2% cholesterol, 46% carbohydrates and 17% casein.	Ration spleen/total body weight and ration liver/total body weight.<br><br>The mean spleen and liver weight in the HFC group was increased compared to chow, but remained similar upon weekly HP-B-CD treatment for a 12-week time period. In line with these data, liver and plasma cholesterol levels were significantly higher upon HFC feeding than after 12 weeks of regular chow. However, no differences in cholesterol concentrations were found between PBS and HP-B-CD-treated mice on an HFC diet. Thus, these data indicate that HP-B-CD has no effect on organ weight and cholesterol concentrations in plasma and liver.
Xie et al. 1999a [28]	BALB/c mice carrying the genetic mutation in the NPC protein were transferred from the National Institutes of Health to the laboratories in Dallas and were used to generate animals that were normal controls (*Npc1*$^{+/+}$) or were either heterozygous (*Npc1*$^{+/-}$) or homozygous (*Npc1*$^{-/-}$) for this genetic defect.	These studies were designed to quantitate the effect of this mutation on whole animal cholesterol turnover, to identify which organs manifested disordered sterol metabolism, to determine if there were disturbances in intracellular cholesterol degradation in the liver and other organs of the live animal, and to identify if there were any abnormalities in cholesterol metabolism in the brain.<br><br>After genotyping and weaning, all animals were fed ad libitum a low cholesterol, pelleted diet (no. 7001; Harlan Teklad, Madison, WI) until they were studied. In one experiment, animals were fed this same diet to which was added 0.2% cholesterol (wt/wt) at the 6th wk of age for 1 wk. The basal diet had a cholesterol content of 0.016% (wt/wt) and a total lipid content of 5% (wt/wt). The principal fatty acids in this basal diet were 0.9% myristic acid, 19.4%	Weight of whole animal, weight of liver, weight of spleen.<br><br>Both the control and homozygous animals had similar food intakes and gained weight at similar rates through the first 7 wk of life. Beyond this time, the *Npc1*$^{-/-}$animals developed neurological findings, had poor food intake, and began to lose weight.<br><br>During this same interval, the relative weight of the liver, spleen, and other organs in the mutant mice progressively increased, a finding reminiscent of the hepatosplenomegaly seen in young children with this disease. In contrast to all of the other organs, however, relative brain weight progressively decreased in the *Npc1*$^{-/-}$mice, reaching only 0.90 of the control weight at 7 wk.

		palmitic acid, 8.6% stearic acid, 31.0% oleic acid, 30.2% linoleic acid, and 2.9% linolenic acid. Most studies were carried out when the mice were 7 wk old, in the fed state, and at the middark phase of the light cycle. In one initial experiment, animals were studied at ages varying from 1 day to 8 wk.	
Xie et al. 1999b [29]	BALByc mice carrying the genetic mutation in Npc1 protein were transferred from the National Institutes of Health.	After weaning, the animals were maintained on a pelleted, basal rodent diet (No. 7001, Harlan Teklad, Madison, WI) that had a cholesterol content of 0.016% (wt/wt) and a total lipid content of 5% (wt/wt). In one experiment, animals were fed for 1 week a meal form of this basal diet, to which was added 0.4% (wt/wt) cholesterol. In most experiments, the animals were studied at 7 weeks of age. In one study, whole animal cholesterol pools were measured in 1-day-old pups.	Weight of whole animal, weight of liver, weight of brain.<br><br>Animal and organ weights, cholesterol pool sizes, and rates of dietary cholesterol absorption in the *Npc1*$^{+/+}$LDLR$^{+/+}$ and *Npc1*$^{-/-}$ LDLR$^{+/+}$mice used in these studies.<br><br>The 7-week-old *Npc1*$^{-/-}$ LDLR$^{+/+}$ animals used in this study had marginally lower body and brain weights and slightly elevated liver weights compared with the *Npc1*$^{+/+}$ LDLR$^{+/+}$ mice. However, after correcting for body weight, brain weight was similar in the mutant (22 g/kg body weight) and control (21 g/kg) animals whereas liver weight was increased 36% (76 g/kg vs. 56 g/kg).
Xie et al. 2000 [30]	Heterozygous *Npc1*$^{+/-}$ mice with a BALB/c background were crossbred with homozygous LDLR knockout (LDLR$^{-/-}$ animals.	After weaning, all animals were housed in plastic colony cages in rooms with alternating 12-h periods of light and dark. All mice were fed a basal rodent diet (No. 7001; Harlan Teklad, Madison, WI) containing 0.016% (w/w) cholesterol until they were studied at 7 weeks of age. In one experiment, either cholesterol (1%, w/w) or cholestyramine (2%, w/w) was added to the meal form of this basal diet. These supplemented diets were begun at the 6th week of age and were fed for 1 week.	Whole body weight, liver weight.<br><br>Animal and organ weights, cholesterol pool sizes, and rates of dietary cholesterol absorption in the *Npc1*$^{+/+}$LDLR$^{+/+}$ and *Npc1*$^{-/-}$ LDLR$^{+/+}$mice used in these studies.<br><br>The whole-body weight and liver weight were significantly lower in the females, compared to the males, of both genotypes.<br><br>Brain size, however, was similar in the two genders.

References

1. Beltroy, E.P.; Richardson, J.A.; Horton, J.D.; Turley, S.D.; Dietschy, J.M. Cholesterol accumulation and liver cell death in mice with Niemann-Pick type C disease. *Hepatology* **2005**, *42*, 886–893, doi:10.1002/hep.20868.

2. Beltroy, E.P.; Liu, B.; Dietschy, J.M.; Turley, S.D. Lysosomal unesterified cholesterol content correlates with liver cell death in murine Niemann-Pick type C disease. *J. Lipid Res.* **2007**, *48*, 869–881, doi:10.1194/jlr.M600488-JLR200.

3. Borbon, I.; Campbell, E.; Ke, W.; Erickson, R.P. The role of decreased levels of Niemann-Pick C1 intracellular cholesterol transport on obesity is reversed in the C57BL/6J,

metabolic syndrome mouse strain: a metabolic or an inflammatory effect? *J. Appl. Genet.* **2012**, *53*, 323–330, doi:10.1007/S13353-012-0099-8.

4. Ebner, L.; Gläser, A.; Bräuer, A.; Witt, M.; Wree, A.; Rolfs, A.; Frank, M.; Vollmar, B.; Kuhla, A. Evaluation of two liver treatment strategies in a mouse model of niemann–pick-disease type C1. *Int. J. Mol. Sci.* **2018**, *19*, 972, doi:10.3390/ijms19040972.

5. Garver, W.S.; Francis, G.A.; Jelinek, D.; Shepherd, G.; Flynn, J.; Castro, G.; Vockley, C.W.; Coppock, D.L.; Pettit, K.M.; Heidenreich, R.A.; et al. The National Niemann-Pick C1 disease database: Report of clinical features and health problems. *Am. J. Med. Genet. Part A* **2007**, *143*, 1204–1211, doi:10.1002/ajmg.a.31735.

6. Hong, S.B.; Seo, M.S.; Park, S.B.; Seo, Y.J.; Kim, J.S.; Kang, K.S. Therapeutic effects of human amniotic epithelial stem cells in Niemann-Pick type C1 mice. *Cytotherapy* **2012**, *14*, 630–638, doi:10.3109/14653249.2012.663485.

7. Jelinek, D.; Heidenreich, R.A.; Erickson, R.P.; Garver, W.S. Decreased Npc1 gene dosage in mice is associated with weight gain. *Obesity* **2010**, *18*, 1457–1459, doi:10.1038/oby.2009.415.

8. Jelinek, D.A.; Maghsoodi, B.; Borbon, I.A.; Hardwick, R.N.; Cherrington, N.J.; Erickson, R.P. Genetic variation in the mouse model of Niemann Pick C1 affects female, as well as male, adiposity, and hepatic bile transporters but has indeterminate effects on caveolae. *Gene* **2012**, *491*, 128–134, doi:10.1016/j.gene.2011.10.010.

9. Jelinek, D.; Castillo, J.J.; Garver, W.S. The C57BL/6J Niemann-Pick C1 mouse model with decreased gene dosage has impaired glucose tolerance independent of body weight. *Gene* **2013**, *527*, 65–70, doi:10.1016/j.gene.2013.05.080.

10. Jiang, D.; Lee, H.; Pardridge, W.M. Plasmid DNA gene therapy of the Niemann-Pick C1 mouse with transferrin receptor-targeted Trojan horse liposomes. *Sci. Rep.* **2020**, *10*, doi:10.1038/s41598-020-70290-w.

11. Li, H.; Repa, J.J.; Valasek, M.A.; Beltroy, E.P.; Turley, S.D.; German, D.C.; Dietschy, J.M. Molecular, anatomical, and biochemical events associated with neurodegeneration in mice with Niemann-Pick type C disease. *J. Neuropathol. Exp. Neurol.* **2005**, *64*, 323–333, doi:10.1093/jnen/64.4.323.

12. Li, H.; Turley, S.D.; Liu, B.; Repa, J.J.; Dietschy, J.M. GM2/GD2 and GM3 gangliosides have no effect on cellular cholesterol pools or turnover in normal or NPC1 mice. *J. Lipid Res.* **2008**, *49*, 1816–1828, doi:10.1194/jlr.M800180-JLR200.

13. Lin, J.; Shao, W.Q.; Chen, Q.Z.; Zhu, W.W.; Lu, L.; Jia, H.L.; Chen, J.H. Osteopontin deficiency protects mice from cholesterol gallstone formation by reducing expression of intestinal NPC1L1. *Mol. Med. Rep.* **2017**, *16*, 1785–1792, doi:10.3892/mmr.2017.6774.

14. Liu, B.; Li, H.; Repa, J.J.; Turley, S.D.; Dietschy, J.M. Genetic variations and treatments that affect the lifespan of the NPC1 mouse. *J. Lipid Res.* **2008**, *49*, 663–9, doi:10.1194/jlr.M700525-JLR200.

15. Lopez, A.M.; Terpack, S.J.; Posey, K.S.; Liu, B.; Ramirez, C.M.; Turley, S.D. Systemic administration of 2-hydroxypropyl-β-cyclodextrin to symptomatic Npc1-deficient mice slows cholesterol sequestration in the major organs and improves liver function. *Clin. Exp. Pharmacol. Physiol.* **2014**, *41*, 780–7, doi:10.1111/1440-1681.12285.

16. Lopez, A.M.; Jones, R.D.; Repa, J.J.; Turley, S.D. Niemann-pick C1-deficient mice lacking sterol O-acyltransferase 2 have less hepatic cholesterol entrapment and improved liver function. *Am. J. Physiol. - Gastrointest. Liver Physiol.* **2018**, *315*, G454–G463, doi:10.1152/ajpgi.00124.2018.

17. Lopez, A.M.; Ramirez, C.M.; Taylor, A.M.; Jones, R.D.; Repa, J.J.; Turley, S.D. Ontogenesis and Modulation of Intestinal Unesterified Cholesterol Sequestration in a Mouse Model of Niemann-Pick C1 Disease. *Dig. Dis. Sci.* **2020**, *65*, 158–167, doi:10.1007/S10620-019-05736-X.

18. Maass, F.; Petersen, J.; Hovakimyan, M.; Schmitt, O.; Witt, M.; Hawlitschka, A.; Lukas, J.; Rolfs, A.; Wree, A. Reduced cerebellar neurodegeneration after combined therapy with cyclodextrin/allopregnanolone and miglustat in NPC1: A mouse model of Niemann-Pick type C1 disease. *J. Neurosci. Res.* **2015**, *93*, 433–442, doi:10.1002/jnr.23509.

19. Mundy, D.I.; Lopez, A.M.; Posey, K.S.; Chuang, J.C.; Ramirez, C.M.; Scherer, P.E.; Turley, S.D. Impact of the loss of caveolin-1 on lung mass and cholesterol metabolism in mice with and without the lysosomal cholesterol transporter, Niemann-Pick type C1. *Biochim. Biophys. Acta - Mol. Cell Biol. Lipids* **2014**, *1841*, 995–1002, doi:10.1016/j.bbalip.2014.04.002.

20. Neßlauer, A.M.; Gläser, A.; Gräler, M.; Engelmann, R.; Müller-Hilke, B.; Frank, M.; Burstein, C.; Rolfs, A.; Neidhardt, J.; Wree, A.; et al. A therapy with miglustat, 2-hydroxypropyl-ß-cyclodextrin and allopregnanolone restores splenic cholesterol homeostasis in Niemann-pick disease type C1. *Lipids Health Dis.* **2019**, *18*, doi:10.1186/s12944-019-1088-2.

21. Parra, J.; Klein, A.D.; Castro, J.; Morales, M.G.; Mosqueira, M.; Valencia, I.; Cortés, V.; Rigotti, A.; Zanlungo, S. Npc1 deficiency in the C57BL/6J genetic background enhances Niemann-Pick disease type C spleen pathology. *Biochem. Biophys. Res. Commun.* **2011**, *413*, 400–406, doi:10.1016/j.bbrc.2011.08.096.

22. Ramirez, C.M.; Liu, B.; Taylor, A.M.; Repa, J.J.; Burns, D.K.; Weinberg, A.G.; Turley, S.D.; Dietschy, J.M. Weekly cyclodextrin administration normalizes cholesterol metabolism in nearly every organ of the Niemann-Pick type C1 mouse and markedly prolongs life. *Pediatr. Res.* **2010**, *68*, 309–15, doi:10.1203/PDR.0b013e3181ee4dd2.

23. Ramirez, C.M.; Lopez, A.M.; Le, L.Q.; Posey, K.S.; Weinberg, A.G.; Turley, S.D. Ontogenic changes in lung cholesterol metabolism, lipid content, and histology in mice with Niemann-Pick type C disease. *Biochim. Biophys. Acta - Mol. Cell Biol. Lipids* **2014**, *1841*, 54–61, doi:10.1016/j.bbalip.2013.09.010.

24. Rodriguez-Gil, J.L.; Watkins-Chow, D.E.; Baxter, L.L.; Yokoyama, T.; Zerfas, P.M.; Starost, M.F.; Gahl, W.A.; Malicdan, M.C. V.; Porter, F.D.; Platt, F.M.; et al. NPC1 deficiency in mice is associated with fetal growth restriction, neonatal lethality and abnormal lung pathology. *J. Clin. Med.* **2020**, *9*, doi:10.3390/jcm9010012.

25. Santiago-Mujica, E.; Flunkert, S.; Rabl, R.; Neddens, J.; Loeffler, T.; Hutter-Paier, B. Hepatic and neuronal phenotype of NPC1 −/− mice. *Heliyon* **2019**, *5*, e01293, doi:10.1016/j.heliyon.2019.e01293.

26. Schlegel, V.; Thieme, M.; Holzmann, C.; Witt, M.; Grittner, U.; Rolfs, A.; Wree, A. Pharmacologic treatment assigned for Niemann Pick Type C1 disease partly changes behavioral traits in wild-type mice. *Int. J. Mol. Sci.* **2016**, *17*, 1866, doi:10.3390/ijms17111866.

27. Walenbergh, S.M.A.; Houben, T.; Hendrikx, T.; Jeurissen, M.L.J.; van Gorp, P.J.; Vaes, N.; Damink, S.W.M.O.; Verheyen, F.; Koek, G.H.; Lütjohann, D.; et al. Weekly treatment of 2-hydroxypropyl-β-cyclodextrin improves intracellular cholesterol levels in LDL receptor knockout mice. *Int. J. Mol. Sci.* **2015**, *16*, 21056–21069, doi:10.3390/ijms160921056.

28. Xie, C.; Turley, S.D.; Pentchev, P.G.; Dietschy, J.M. Cholesterol balance and metabolism in mice with loss of function of Niemann-Pick C protein. *Am. J. Physiol. - Endocrinol. Metab.* **1999**, *276*, doi:10.1152/ajpendo.1999.276.2.e336.

29. Xie, C.; Turley, S.D.; Dietschy, J.M. Cholesterol accumulation in tissues of the Niemann-Pick type C mouse is determined by the rate of lipoprotein-cholesterol uptake through the coated-pit pathway in each organ. *Proc. Natl. Acad. Sci. U. S. A.* **1999**, *96*, 11992–11997, doi:10.1073/pnas.96.21.11992.

30. Xie, C.; Turley, S.D.; Dietschy, J.M. Centripetal cholesterol flow from the extrahepatic organs through the liver is normal in mice with mutated Niemann-Pick type C protein (NPC1). *J. Lipid Res.* **2000**, *41*, 1278–1289, doi:10.1016/S0022-2275(20)33436-2.
